# The solid effect of dynamic nuclear polarization in liquids – accounting for *g*-tensor anisotropy at high magnetic fields

**DOI:** 10.5194/mr-4-243-2023

**Published:** 2023-11-01

**Authors:** Deniz Sezer, Danhua Dai, Thomas F. Prisner

**Affiliations:** Institute of Physical and Theoretical Chemistry, Goethe University, 60438 Frankfurt am Main, Germany

## Abstract

In spite of its name, the solid effect of dynamic nuclear polarization (DNP) is also operative in viscous liquids, where the dipolar interaction between the polarized nuclear spins and the polarizing electrons is not completely averaged out by molecular diffusion on the timescale of the electronic spin–spin relaxation time. Under such slow-motional conditions, it is likely that the tumbling of the polarizing agent is similarly too slow to efficiently average the anisotropies of its magnetic tensors on the timescale of the electronic $T_{2}$ . Here we extend our previous analysis of the solid effect in liquids to account for the effect of g -tensor anisotropy at high magnetic fields. Building directly on the mathematical treatment of slow tumbling in electron spin resonance [Bibr bib1.bibx14], we calculate solid-effect DNP enhancements in the presence of both translational diffusion of the liquid molecules and rotational diffusion of the polarizing agent. To illustrate the formalism, we analyze high-field (9.4 T) DNP enhancement profiles from nitroxide-labeled lipids in fluid lipid bilayers. By properly accounting for power broadening and motional broadening, we successfully decompose the measured DNP enhancements into their separate contributions from the solid and Overhauser effects.

## 1 Introduction

The sensitivity of NMR experiments is greatly increased by dynamic nuclear polarization (DNP)Abbreviations used in the text: 1,3-bisdiphenylene-2-phenylallyl (BDPA), continuous wave (cw), 1,2-dimyristoyl-sn-glycero-3-phosphocholine (DMPC), 1,2-dioleoyl-sn-glycero-3-phosphocholine (DOPC), double quantum (DQ), dynamic nuclear polarization (DNP), electron paramagnetic resonance (EPR), force-free hard sphere (FFHS), microscopic order macroscopic disorder (MOMD), microwave (mw), nuclear magnetic resonance (NMR), Overhauser effect (OE), 1-palmitoyl-2-stearoyl-sn-glycero-3-phosphocholine (PSPC), solid effect (SE), stochastic Liouville equation (SLE), slowly relaxing local structure (SRLS), 4-Hydroxy-2,2,6,6-tetramethylpiperidin-1-oxyl (TEMPOL), zero quantum (ZQ)., where the much larger static polarization that is available to electronic spins is transferred to nuclear spins [Bibr bib1.bibx5]. For the transfer to take place, the electronic and nuclear spins should be able to flip simultaneously [Bibr bib1.bibx2]. Such concerted flips correspond to the zero-quantum (ZQ) and double-quantum (DQ) transitions of the electron–nucleus spin system, which are enabled by inter-spin interactions. Among the four DNP mechanisms, i.e., the Overhauser effect (OE), the solid effect (SE), the cross effect and thermal mixing, only the first two have been conclusively shown to be operative in the liquid state, where the spin–spin interactions change randomly in time due to the thermal motions of the molecules.

In OE-DNP, the ZQ and DQ transitions are in fact possible because the dipole–dipole and contact interactions are modulated by molecular motions. In SE-DNP, on the other hand, the ZQ and DQ transitions are driven directly by mw excitation, and the modulation of the dipolar interaction is detrimental because it constantly modifies the matching condition that the mw frequency should satisfy in order to resonantly drive these transitions.

The initial theoretical treatments of OE [Bibr bib1.bibx48] and SE [Bibr bib1.bibx3] modeled the ZQ and DQ transitions by expressing the transition probabilities per unit time using Fermi's golden rule. As the mathematical description of (semi-classical) relaxation theory matured around the same time [Bibr bib1.bibx41], Fermi's golden rule was promptly replaced in the theory of OE-DNP in liquids [Bibr bib1.bibx19] with the correlation function of the dipolar interaction (or its Laplace transform, which is known as spectral density). Because the time-domain description of relaxation leads to a correlation function in a very general way [Bibr bib1.bibx1], the same formalism works naturally with different spectral densities (e.g., for rotational or translational diffusion). As an example, the improved analytical treatment of isotropic translational diffusion achieved in 1975 was immediately applied to paramagnetic relaxation in liquids [Bibr bib1.bibx6].Surprisingly, this improved treatment is not mentioned by [Bibr bib1.bibx34], who continue to use the older, deficient expression of spectral density for translational diffusion.


During the same time period, it also became possible to account for spin dephasing and relaxation beyond second order [Bibr bib1.bibx4], which is important for understanding spectral line shapes outside the regime of fast averaging [Bibr bib1.bibx22]. These initial ideas were transformed into a powerful tool for the calculation and analysis of slow-motional EPR spectra by Freed [Bibr bib1.bibx14].

When first presented, Abragam's quantitative description of SE-DNP in terms of mixing of the Zeeman energy levels by the dipolar interaction [Bibr bib1.bibx3] conclusively explained that the NMR signal is maximally enhanced when the mw frequency is shifted from the electronic resonance by $\pm \omega_{I}$ , where $\omega_{I}$ is the Larmor frequency of the polarized nuclear spin. Abragam's perturbative analysis also correctly predicted that the effect should drop quadratically with the magnitude of the static magnetic field, which has lasting implications for SE-DNP at high magnetic fields. In spite of these successes, however, the perturbative approach to SE is practically impossible to integrate with other relevant spin phenomena whose mathematical treatment matured subsequently.

Recently, [Bibr bib1.bibx44] presented a time-domain description of SE which, like semi-classical relaxation theory, allows for different dynamical processes to modulate the relevant spin interactions. By interfacing this description with the spectral density of isotropic translational diffusion [Bibr bib1.bibx6], it was possible to treat SE-DNP in the presence of molecular translation as relevant to homogeneous liquids [Bibr bib1.bibx45]. The requirement that the dipolar interaction should not be completely averaged out by the molecular dynamics during the electronic $T_{2}$ restricts liquid-state SE-DNP to viscous media, where the tumbling of the polarizing agent may similarly be too slow to average the anisotropies of its magnetic tensors. The current paper accounts for the effect of g -tensor anisotropy on SE in this slow-tumbling regime. To this end, the time-domain description of SE-DNP in liquids is interfaced here with the established mathematical treatment of slow-motional EPR spectra [Bibr bib1.bibx14]. For the illustrative purposes of the current paper, we only consider free (i.e., unrestricted) rotational diffusion with an isotropic diffusion coefficient. Nevertheless, the treatment can be analogously extended to anisotropic diffusion in an orienting potential by building on the general mathematical formalism of the MOMD (microscopic order macroscopic disorder) and SRLS (slowly relaxing local structure) models [Bibr bib1.bibx33].

To motivate the presented theoretical analysis, in Sect. [Sec Ch1.S2] we formulate one specific practical problem that it addresses. There we also introduce the experimental EPR and DNP data that are analyzed subsequently in Sect. [Sec Ch1.S5] using the developed theory. The needed background from [Bibr bib1.bibx44] is presented in Sect. [Sec Ch1.S3]. Building on this, in Sect. [Sec Ch1.S4] we adapt the slow-motional formalism of [Bibr bib1.bibx14] to the treatment of SE in the liquid state. Our conclusions are in Sect. [Sec Ch1.S6], and several supporting figures are left to the Appendix.

## 2 Motivation

DNP aims to increase the longitudinal nuclear magnetization, $i_{z}$ , beyond its equilibrium Boltzmann value, $i_{z}^{\mathrm{eq}}$ . This is done by doping the sample with unpaired electrons, whose spins are then subjected to near-resonance mw irradiation. In cw-DNP, which is the only variety that we consider here, a steady-state magnetization $i_{z}^{\mathrm{ss}}$ is reached after the microwaves have been applied for a sufficiently long time. The enhancement of $i_{z}$ under such steady-state conditions is

1 $$\epsilon =\frac{i_{z}^{\mathrm{ss}}}{i_{z}^{\mathrm{eq}}}-1,$$

where ϵ=0 corresponds to the absence of DNP.

In both OE and SE, ϵ is directly proportional to the ratio of the gyromagnetic factors of the electronic and nuclear spins, $\gamma_{S}$ and $\gamma_{I}$ . For OE [Bibr bib1.bibx19],

2 $$\epsilon_{\mathrm{OE}}=scf\frac{\left|\gamma_{S}\right|}{\gamma_{I}},$$

where s , c and f are, respectively, the electronic saturation factor, the coupling factor and the leakage factor. The first is defined as

3 $$s=1-s_{z}^{\mathrm{ss}}/s_{z}^{\mathrm{eq}}$$

and reflects the deviation of the longitudinal electronic magnetization at steady state, $s_{z}^{\mathrm{ss}}$ , from its equilibrium value, $s_{z}^{\mathrm{eq}}$ . The other two factors, c and f , quantify the interaction between the electronic and nuclear spins. Specifically, the leakage factor

4 $$f=1-T_{1I}/T_{1I}^{0}$$

compares the nuclear $T_{1}$ s in the presence ( $T_{1I}$ ) and absence ( $T_{1I}^{0}$ ) of the polarizing agent. In DNP, $T_{1I}$ is typically (much) shorter than $T_{1I}^{0}$ due to the elevated concentration of the electronic spins, and hence f≈1 .

Similarly, the SE enhancement can be expressed as [Bibr bib1.bibx44]

5 $$\epsilon_{\mathrm{SE}}=pv_{-}T_{1I}\left(\frac{1}{1+v_{+}T_{1I}}\right)\frac{\left|\gamma_{S}\right|}{\gamma_{I}},$$

where p=1-s quantifies how “non-saturated” the electronic transition is, and the rate constants $v_{+}$ and $v_{-}$ are related to the ability of the microwaves to excite simultaneous flips of the electronic and nuclear spins. These concerted flips correspond to the “forbidden” ZQ and DQ transitions, which are enabled by the dipolar interaction. In fact,

6 $$v_{\pm }=v_{2}\pm v_{0},$$

where $v_{0}$ and $v_{2}$ denote, respectively, the ZQ and DQ transition rate constants. In liquids, where the dipolar interaction is partially averaged, the contribution of the mw excitation to the nuclear relaxation rate $R_{1I}=1/T_{1I}$ , which is quantified by $v_{+}$ , is generally negligibly small. As a result, $v_{+}/R_{1I}\ll 1$ , and the expression in parentheses in Eq. ([Disp-formula Ch1.E5]) is essentially 1. Then the SE enhancement acquires the following multiplicative form:

7 $$\epsilon_{\mathrm{SE}}\approx pv_{-}T_{1I}\frac{\left|\gamma_{S}\right|}{\gamma_{I}}\;(v_{+}\ll R_{1I}),$$

which is analogous to $\epsilon_{\mathrm{OE}}$ with the factors s , c and f being replaced with the factors p , $v_{-}$ and $T_{1I}$ , respectively. In the numerical work presented in Sect. [Sec Ch1.S5] we use the approximation in Eq. ([Disp-formula Ch1.E7]). The condition $v_{+}T_{1I}\ll 1$ is validated at the end of the analysis by comparing the estimated $v_{+}$ to the measured $T_{1I}$ .

In the current paper we study the dependence of the DNP enhancement on the displacement from the electronic resonance. Following [Bibr bib1.bibx17], we call the profile of ϵ against the offset from resonance a “DNP spectrum”. Because DNP experiments in the liquid state are carried out with a mw resonator [Bibr bib1.bibx10], off-resonance conditions are achieved by varying the stationary magnetic field at a constant mw frequency (i.e., field sweep). In theoretical analysis, however, it is more convenient to work with a fixed $B_{0}$ and a variable mw frequency. Thus, when comparing calculations and experiments, we will convert the horizontal axis of the experiments from units of magnetic field to units of offset frequency.

In the case of $\epsilon_{\mathrm{OE}}$ (Eq. [Disp-formula Ch1.E2]), the entire offset dependence is due to the saturation factor s , as the factors c and f are practically constant over such a narrow frequency range. In the case of $\epsilon_{\mathrm{SE}}$ (Eq. [Disp-formula Ch1.E5]), both $pv_{-}$ and $v_{+}$ are functions of the offset. For a single, homogeneously broadened EPR line the saturation factor can be obtained in closed analytical form from the Bloch equations (as we review below in Sect. [Sec Ch1.S3.SS1]). Recently, [Bibr bib1.bibx44] showed that the SE spin dynamics is described by two coupled Bloch equations, whose steady state can similarly be solved analytically to obtain closed-form expressions for the rate constants $v_{\pm }$ (reviewed in Sect. [Sec Ch1.S3.SS2]). In liquids, where the random molecular motion modulates the dipolar interaction between the electronic and nuclear spins, these rate constants are no longer available analytically but can be calculated numerically for motional models with known dipolar spectral densities [Bibr bib1.bibx45], as reviewed below in Sect. [Sec Ch1.S3.SS3].

Liquid-state SE-DNP is restricted to viscous media, where the dipolar interaction is not averaged out completely on the decoherence timescale of the electronic spins. Under these conditions, the tumbling of the polarizing agent is also expected to be too slow to average the anisotropies of its magnetic tensors on the timescale of the electronic $T_{2}$ . One thus expects substantial deviations from the Lorentzian EPR line shape of the Bloch equations. Such deviations are unavoidable in the case of nitroxide-based polarizing agents whose g and A tensors are rather anisotropic. A recent SE-DNP study at 9.4 T demonstrated that even the narrow-line radical trityl exhibited g -tensor broadening in liquid glycerol [Bibr bib1.bibx26].

This paper extends the theoretical description of SE-DNP to the regime of slow radical tumbling, where the cw-EPR line shape is not Lorentzian. Given our long-standing efforts in liquid-state DNP at 9.4 T, here we focus on high magnetic fields, where the width of the EPR spectrum is dominated by the anisotropy of the g tensor. We will thus completely neglect the hyperfine tensor. This possibility greatly simplifies the needed adjustments to the Lorentzian case (Sect. [Sec Ch1.S4]).

To illustrate the practical problem that motivated this theoretical work, we now turn to the experimental data in Fig. [Fig Ch1.F1]. The characterized samples comprised liposomes of hydrated lipid bilayers composed of DOPC (1,2-dioleoyl-sn-glycero-3-phosphocholine) lipids. As the phase transition temperature of DOPC is about -17 ${}^{\circ }$ C, the lipids were in their fluid, liquid-crystalline phase in the experiments at ≈320 K. The DOPC lipids were mixed at a ratio of 20:1 with PSPC lipids spin-labeled either at position 10 (1-palmitoyl-2-stearoyl-(10-doxyl)-sn-glycero-3-phosphocholine) or at position 16 along one of their aliphatic chains. Both the EPR spectra (Fig. [Fig Ch1.F1]a, b) and the DNP enhancements (Fig. [Fig Ch1.F1]c, d) were recorded in our home-built Fabry–Pérot resonator at 9.4 T equipped with a temperature control [Bibr bib1.bibx9]. While the target temperature of the experiments was 320 K, an extra temperature rise of less than 10 ${}^{\circ }$ C can be expected at the maximum mw power of 5.5 W that was used for DNP [Bibr bib1.bibx9]. Details about the experiments and the sample preparation will be published elsewhere.

**Figure 1 Ch1.F1:**
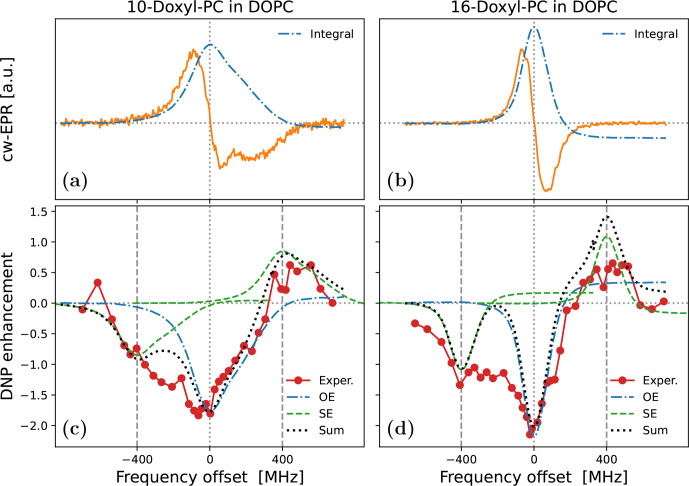
Experimental cw-EPR spectra **(a, b)** and DNP spectra **(c, d)** of spin-labeled lipids in DOPC lipid bilayers at 9.4 T and ≈320 K. The nitroxide spin label (Doxyl) is either at position 10 **(a, c)** or at position 16 **(b, d)** of the aliphatic lipid chain. The integrated cw-EPR spectra (dashed–dotted blue lines in panels **a** and **b**) are used to decompose the DNP spectra (**c** and **d**) into contributions from OE (dashed–dotted blue lines) and SE (dashed green lines). The sum of these two contributions is indicated with a dotted black line.

The cw-EPR spectrum of 10-Doxyl-PC in Fig. [Fig Ch1.F1]a (orange line) is seen to deviate substantially from (the derivative of) a Lorentzian line shape. At this high magnetic field, the EPR line width is expected to be dominated by the large anisotropy of the nitroxide g tensor, with a comparatively much smaller contribution from the nitroxide hyperfine tensor. (These expectations are tested and verified below in Sect. [Sec Ch1.S5.SS1].) For comparison, in Fig. [Fig Ch1.F1]b we show the cw-EPR spectrum of the sample doped with 16-Doxyl-PC. Visually, this narrower spectrum more closely resembles a homogeneous Lorentzian line, although it still deviates from it (as discussed in Sect. [Sec Ch1.S5.SS2]).

In Fig. [Fig Ch1.F1]c we show the DNP spectrum (filled red circles) of the sample containing 10-Doxyl-PC as a polarizing agent. The enhanced NMR signal belongs to the acyl chain protons of the lipids. Thanks to the high magnetic field of the experiment, it was possible to resolve the NMR signal of these non-polar protons from the polar protons of water and of the lipid head groups. The DNP spectrum is seen to have a complex line shape, with positive enhancement values at offsets of about +400 MHz demonstrating a contribution from SE. At the same time, the comparatively larger negative enhancements in the vicinity of the electronic resonance (i.e., around 0 MHz) point to a contribution from OE. Such coexistence of SE and OE is well documented for nitroxide free radicals at the classical EPR fields of about 0.35 T [Bibr bib1.bibx28]. Evidently, it also persists at 9.4 T. The DNP spectrum of 16-Doxyl-PC in Fig. [Fig Ch1.F1]d also exhibits a mixture of SE and OE.

More than half a century ago, Korringa and coworkers developed a rigorous theoretical framework to predict such mixed DNP spectra in viscous liquids [Bibr bib1.bibx38]. Likely because of its complexity and its neglect of translational diffusion, their formal analysis has not been applied to recent DNP data. As a simple and practical alternative, [Bibr bib1.bibx36] disentangled the OE and SE components of such mixed DNP spectra using only the integral of the measured cw-EPR signal. Their approach is based on the following insightful observations: (i) up to an overall scaling factor, the EPR line shape is equal to the saturation factor and thus to the OE enhancement (Eq. [Disp-formula Ch1.E2]); (ii) up to an overall scaling factor, the SE enhancement lines at $\pm \omega_{I}$ are shifted versions (and flipped for the ZQ transition) of the same EPR line shape. One can thus identify the contributions of OE and SE to the DNP spectrum by placing the integrated cw-EPR spectrum at, respectively, zero and $\pm \omega_{I}$ offsets and independently adjusting the magnitudes of the two components.

This approach is illustrated in Fig. [Fig Ch1.F1]c and [Fig Ch1.F1]d, where the dashed–dotted blue lines are the integrals of the cw-EPR spectra from Fig. [Fig Ch1.F1]a and [Fig Ch1.F1]b, respectively (flipped here to reflect the dipolar nature of OE), and the dashed green lines are the same EPR spectra but centered at -400 and +400 MHz. The sum of the OE and SE contributions determined in this way is shown with a dotted black line. This sum is seen to agree closely with the DNP spectrum of 10-Doxyl-PC (Fig. [Fig Ch1.F1]c) and to capture well the overall shape of the DNP spectrum of 16-Doxyl-PC (Fig. [Fig Ch1.F1]d).

In spite of the good general agreement between the experimental DNP spectra and the dotted black lines in Fig. [Fig Ch1.F1]c and [Fig Ch1.F1]d, some persistent differences remain. In particular, (i) the OE feature in the experiment appears to be consistently broader than the EPR line and (ii) the enhancement between the central OE feature and the negative SE feature is consistently larger than what is predicted by the overlap of the two copies of the EPR line shape. Both of these aspects are especially clear in the case of 16-Doxyl-PC (Fig. [Fig Ch1.F1]d). The theory presented below (Sect. [Sec Ch1.S4]) aims to address these deficiencies of the simple approach.

In fact, the first deficiency is easy to rationalize. Cw-EPR spectra are recorded at low mw power, and their widths reflect mechanisms contributing to the electronic $T_{2}$ relaxation. The DNP spectrum, on the other hand, is recorded at high mw power, where the EPR line width experiences power broadening that also depends on the electronic $T_{1}$ relaxation. That the OE-DNP spectrum “represents an indirect observation of the electron resonance when greatly saturated” was understood early on ([Bibr bib1.bibx7], Fig. 6). To properly model the contribution of OE to mixed DNP spectra, therefore, it is necessary to calculate the cw-EPR spectrum under saturating conditions. How to rigorously do that in the regime of slow radical tumbling is known [Bibr bib1.bibx14].

While power broadening affects OE, it is not immediately clear whether one should also take it into account when modeling SE. (We address this point in Sect. [Sec Ch1.S4.SS4].) Even leaving power broadening aside, however, we know that in liquids the SE lines of the DNP spectrum should also be broader than the EPR line width because of the fluctuations of the dipolar interaction [Bibr bib1.bibx45]. Although [Bibr bib1.bibx45] showed how to quantify this additional motional broadening in the case of translational molecular diffusion, the theoretical treatment there assumed a Lorentzian EPR line and is thus not directly applicable to the experiments in Fig. [Fig Ch1.F1]. In the current paper, we extend the formalism to slow radical tumbling and g -tensor anisotropy (Sect. [Sec Ch1.S4]). In Sect. [Sec Ch1.S5] we apply the developed theory to the analysis of the experimental spectra in Fig. [Fig Ch1.F1], disentangling the contributions of SE and OE to the observed DNP. The needed theoretical background from [Bibr bib1.bibx44] is reviewed next.

## 3 Theoretical background

The classical Bloch equations describe the dynamics of the electronic magnetization, including under saturating conditions. In Sect. [Sec Ch1.S3.SS1] we recall the relationship between the steady-state solution of the Bloch equations and cw-EPR. Then, in Sect. [Sec Ch1.S3.SS2], closed-form expressions are obtained for the rate constants of the forbidden transitions that are driven by the microwaves in SE-DNP. These expressions, derived in this form for the first time (Eqs. [Disp-formula Ch1.E33] and [Disp-formula Ch1.E34]), are similar to the steady-state solutions of the Bloch equations but additionally contain (i) the strength of the electron–nucleus dipolar interaction and (ii) the Larmor frequency of the polarized nuclear spin [Bibr bib1.bibx44]. Finally, in Sect. [Sec Ch1.S3.SS3] we remind the reader how these expressions should be modified in the presence of random modulation of the dipolar interaction, as relevant for liquids [Bibr bib1.bibx45].

The reviewed results, which apply to a single Lorentzian line, will be extended in Sect. [Sec Ch1.S4] to the regime of slow radical tumbling and an anisotropic g tensor. In the process, some of the scalar variables that appear below, like the offset frequency and the electronic relaxation rates, will be replaced with square matrices, as we explain in Sect. [Sec Ch1.S4.SS1] and [Sec Ch1.S4.SS2]. The generalization of Sect. [Sec Ch1.S3.SS1], [Sec Ch1.S3.SS2] and [Sec Ch1.S3.SS3] along these lines is carried out in, respectively, Sect. [Sec Ch1.S4.SS3], [Sec Ch1.S4.SS4] and [Sec Ch1.S4.SS5].

### 3.1 Bloch equations

The evolution of the expectation values of the electronic spin operators $S_{i}$ ( i=x,y,z ), which we denote by $s_{i}$ , is described by the classical Bloch equations (in the rotating frame)

8 $$\left[\begin{array}{c} {\dot{s}}_{x}(t)\\ {\dot{s}}_{y}(t)\\ {\dot{s}}_{z}(t) \end{array}\right]=-\left[\begin{array}{ccc} R_{2} & \Delta & 0\\ -\Delta & R_{2} & \omega_{1}\\ 0 & -\omega_{1} & R_{1} \end{array}\right]\left[\begin{array}{c} s_{x}(t)\\ s_{y}(t)\\ s_{z}(t) \end{array}\right]+R_{1}\left[\begin{array}{c} 0\\ 0\\ s_{z}^{\mathrm{eq}} \end{array}\right].$$

Here, the dot above the variable indicates differentiation with respect to time, $R_{2}$ and $R_{1}$ are the reciprocals of the electronic relaxation times $T_{2}$ and $T_{1}$ , respectively, andIn [Bibr bib1.bibx44], the frequency offset was denoted by Ω . Here we denote the offset by Δ and reserve the symbol Ω for the orientation of the polarizing agent (Sect. [Sec Ch1.S4]).

9 $$\Delta =\omega_{0}-\omega$$

is the offset between the Larmor frequency of the electronic spins, $\omega_{0}$ , and the (angular) frequency of the oscillating magnetic field, ω . In the case of an isotropic g factor, $g_{0}$ ,

10 $$\omega_{0}=g_{0}\mu_{B}B_{0}/\hbar ,$$

where $\mu_{B}$ is the Bohr magneton and ℏ is the reduced Planck constant.

At steady state,

11 $$\left[\begin{array}{ccc} R_{2} & \Delta & 0\\ -\Delta & R_{2} & \omega_{1}\\ 0 & -\omega_{1} & R_{1} \end{array}\right]\left[\begin{array}{c} s_{x}^{\mathrm{ss}}\\ s_{y}^{\mathrm{ss}}\\ s_{z}^{\mathrm{ss}} \end{array}\right]=R_{1}\left[\begin{array}{c} 0\\ 0\\ s_{z}^{\mathrm{eq}} \end{array}\right].$$

Solving these algebraic equations for the variables $s_{i}^{\mathrm{ss}}$ , one can calculate the cw-EPR spectrum and the electronic saturation profile. Making use of the zeros in the first and last rows of the Bloch matrix in Eq. ([Disp-formula Ch1.E11]), we first express $s_{x}^{\mathrm{ss}}$ and $s_{z}^{\mathrm{ss}}$ in terms of $s_{y}^{\mathrm{ss}}$ :

12 $$s_{x}^{\mathrm{ss}}=-\Delta T_{2}s_{y}^{\mathrm{ss}},\;s_{z}^{\mathrm{ss}}=\omega_{1}T_{1}s_{y}^{\mathrm{ss}}+s_{z}^{\mathrm{eq}}.$$

The middle row of the matrix then yields

13 $$s_{y}^{\mathrm{ss}}=-\omega_{1}P_{0}^{-1}s_{z}^{\mathrm{eq}},$$

where we defined

14 $$P_{0}=R_{2}+\omega_{1}^{2}T_{1}+\Delta^{2}T_{2}.$$

The in-phase (absorptive) and out-of-phase (dispersive) components of the cw-EPR signal are then found to be

15 $$\begin{array}{rl} \mathrm{abs} & =s_{y}^{\mathrm{ss}}/s_{z}^{\mathrm{eq}}=-\omega_{1}P_{0}^{-1},\\ \mathrm{dsp} & =s_{x}^{\mathrm{ss}}/s_{z}^{\mathrm{eq}}=-\Delta T_{2}\;\mathrm{abs}. \end{array}$$

From the longitudinal component at steady state, we similarly find

16 $$s=1-s_{z}^{\mathrm{ss}}/s_{z}^{\mathrm{eq}}=-\omega_{1}T_{1}\;\mathrm{abs},$$

which shows that the saturation factor is directly proportional to the absorptive EPR line shape. This proportionality holds for all mw powers, including the large powers used in DNP. In Sect. [Sec Ch1.S4.SS3] we show that this also remains valid in the case of g -tensor anisotropy and isotropic rotational diffusion.

When generalizing the Bloch equations to an anisotropic g tensor, we will need to work with high-dimensional abstract vectors. To distinguish these vectors from the vectors in 3D space, we will denote the latter by placing an arrow above their symbols and will use bold symbols for the former. (A 3D unit vector will be indicated with a hat rather than an arrow.) Additionally, we will use capital hollow letters to denote 3×3 matrices that act on the 3D vectors. With this understanding, we will write the Bloch equations in Eq. ([Disp-formula Ch1.E8]) as

17 $$\vec{\dot{s}}(t)=-B_{0}\vec{s}(t)+R_{1}\hat{k}s_{z}^{\mathrm{eq}},$$

where

18 $$\vec{s}(t)=\left[\begin{array}{c} s_{x}(t)\\ s_{y}(t)\\ s_{z}(t) \end{array}\right],\;\hat{k}=\left[\begin{array}{c} 0\\ 0\\ 1 \end{array}\right],$$

19 $$B=\left[\begin{array}{ccc} R_{2}+\text{i}\omega_{I} & \Delta & 0\\ -\Delta & R_{2}+\text{i}\omega_{I} & \omega_{1}\\ 0 & -\omega_{1} & R_{1}+\text{i}\omega_{I} \end{array}\right],$$

and $B_{0}=B(\omega_{I}=0)$ . The $\text{i}\omega_{I}$ that has been added to the main diagonal of the matrix B will be needed for the dynamical description of the solid effect (see Sect. [Sec Ch1.S3.SS2]). The subscript of $B_{0}$ is intended as a reminder that B is evaluated at $\omega_{I}=0$ , where $\omega_{I}$ is the Larmor frequency of the polarized nuclear spin.

### 3.2 Solid effect in solids

SE relies on the dipolar interaction between the electronic and nuclear spins whose coupling is

20 $$A_{1}=D_{\mathrm{dip}}\frac{-3\mathrm{cos}\theta \mathrm{sin}\theta }{r^{3}}{\text{e}}^{\text{i}\phi }.$$

Here $D_{\mathrm{dip}}=(\mu_{0}/4\pi )\hbar \gamma_{S}\gamma_{I}$ is the dipolar constant, which equals approximately 2π ( 79 kHz nm ${}^{3}$ ) for protons, and (r,θ,ϕ) are the spherical polar coordinates of the inter-spin vector.

In liquids, $A_{1}$ changes in time because of molecular diffusion. The treatment of SE-DNP for a time-dependent $A_{1}$ in [Bibr bib1.bibx45] was developed under the assumption that the nuclear $T_{1}$ is orders of magnitude larger than the correlation time of the electron–nucleus dipolar interaction, which is practically always the case in liquids. For the same analysis to apply to solids, nuclear spin diffusion, which analogously to molecular diffusion in liquids spreads out the nuclear polarization across the sample, should be much faster than the nuclear $T_{1}$ . Although this condition is not necessarily satisfied in the solid state, for the mathematical description in terms of a dipolar correlation function to apply, we will assume that spin diffusion is fast when referring to solids. Similarly, when accounting for g -tensor anisotropy below, we will assume that the tumbling of the radical is much faster than the nuclear $T_{1}$ . This assumption is clearly violated in solids, where “tumbling” is infinitely slow. Nevertheless, for the purposes of comparison, we will refer in the following to “solids” with the understanding that the correlation time of the dipolar interaction is much shorter than the nuclear $T_{1}$ (in order to treat nuclear spin diffusion on the level of a translational correlation function) but much longer than all other relaxation timescales (in order to treat the electron–nucleus dipolar interaction as constant). Because we will keep all other parameters, including the timescale of radical tumbling, the same when comparing “solids” and liquids, it should be kept in mind that our treatment is not a good model for the solid state (hence the quotation marks).

For SE-DNP, in addition to the Bloch equations, it is necessary to consider the following dynamical equations of the electron–nucleus coherences $g_{i}=\langle S_{i}I_{+}\rangle$ ( i=x,y,z ) [Bibr bib1.bibx44]:

21 $$\begin{array}{rl} \left[\begin{array}{c} {\dot{g}}_{x}(t)\\ {\dot{g}}_{y}(t)\\ {\dot{g}}_{z}(t) \end{array}\right] & =-B\left[\begin{array}{c} g_{x}(t)\\ g_{y}(t)\\ g_{z}(t) \end{array}\right]-\frac{1}{4}A_{1}\left[\begin{array}{c} s_{y}(t)\\ -s_{x}(t)\\ 0 \end{array}\right]\\ & -i\frac{1}{4}A_{1}\left[\begin{array}{c} 0\\ 0\\ i_{z}(t) \end{array}\right]. \end{array}$$

Again, we are only interested in the steady state of the dynamics where

22 $$B\left[\begin{array}{c} g_{x}^{\mathrm{ss}}\\ g_{y}^{\mathrm{ss}}\\ g_{z}^{\mathrm{ss}} \end{array}\right]=-\frac{1}{4}A_{1}\left[\begin{array}{c} s_{y}^{\mathrm{ss}}\\ -s_{x}^{\mathrm{ss}}\\ 0 \end{array}\right]-i\frac{1}{4}A_{1}\left[\begin{array}{c} 0\\ 0\\ i_{z}^{\mathrm{ss}} \end{array}\right].$$

The rate constants $pv_{-}$ and $v_{+}$ needed to calculate the SE enhancement (Eq. [Disp-formula Ch1.E5]) are determined from $g_{z}^{\mathrm{ss}}$ using the following equality, which combines [Bibr bib1.bibx44] (2023a, Eq. 31) and [Bibr bib1.bibx45] (2023b, Eq. 42):

23 $${\dot{i}}_{z}{|}_{\mathrm{coh}}^{\mathrm{ss}}=-\text{Re}\{\text{i}A_{1}^{*}g_{z}^{\mathrm{ss}}\}=-R_{1I}^{A}i_{z}^{\mathrm{ss}}-v_{+}i_{z}^{\mathrm{ss}}-pv_{-}s_{z}^{\mathrm{eq}}.$$

( Re{} takes the real part of its argument.) The term proportional to $R_{1I}^{A}$ on the right-hand side of Eq. ([Disp-formula Ch1.E23]) accounts for the contribution of the coherences $g_{i}$ to the nuclear $T_{1}$ relaxation in the absence of mw excitation. This contribution should be removed when calculating the mw-related rates $v_{+}$ and $pv_{-}$ .

To a good approximation, the electronic spin dynamics is independent of the dipolar interaction with the nuclear spins, as other mechanisms are more efficient at causing electronic relaxation, especially in liquids. As a result, the steady-state expressions from Sect. [Sec Ch1.S3.SS1] can be used when solving Eq. ([Disp-formula Ch1.E22]) for $g_{z}^{\mathrm{ss}}$ .

Inverting the matrix B in Eq. ([Disp-formula Ch1.E22]) and using $s_{x,y}^{\mathrm{ss}}$ from before, we find

24 $$\begin{array}{rl} g_{z}^{\mathrm{ss}} & =\omega_{1}\frac{1}{4}A_{1}([B^{-1}{]}_{zx}+\Delta T_{2}[B^{-1}{]}_{zy})P_{0}^{-1}s_{z}^{\mathrm{eq}}\\ & \;-\text{i}\frac{1}{4}A_{1}[B^{-1}{]}_{zz}i_{z}^{\mathrm{ss}}, \end{array}$$

where $[B^{-1}{]}_{ij}$ is the ij th matrix element of $B^{-1}$ . Substituting this $g_{z}^{\mathrm{ss}}$ into Eq. ([Disp-formula Ch1.E23]), we identify the desired SE rate constants

25 $$\begin{array}{rl} R_{1I}^{A} & =\delta^{2}\text{Re}\{[B_{\omega_{1}=0}^{-1}{]}_{zz}\},\\ v_{+} & =\delta^{2}\text{Re}\{[B^{-1}{]}_{zz}\}-R_{1I}^{A},\\ pv_{-} & =-\delta^{2}\omega_{1}P_{0}^{-1}\text{Im}\{[B^{-1}{]}_{zx}+\Delta T_{2}[B^{-1}{]}_{zy}\}, \end{array}$$

where

26 $$\delta^{2}=(A_{1}^{*}A_{1})/4$$

reflects the strength of the dipolar interaction. ( Im{} takes the imaginary part of its argument.)

In liquids, where $A_{1}$ is time-dependent, we will need to modify the matrix $B^{-1}$ in Eq. ([Disp-formula Ch1.E25]) without changing the structure of these expressions (Sect. [Sec Ch1.S3.SS3]). In the case of solids (i.e., when $A_{1}$ does not change with time), it is possible to carry out the inversion of B by expressing $g_{x}^{\mathrm{ss}}$ and $g_{z}^{\mathrm{ss}}$ in terms of $g_{y}^{\mathrm{ss}}$ , analogously to our treatment of the Bloch equations in the previous subsection.

From the upper and lower rows of B in Eq. ([Disp-formula Ch1.E22]), we find

27 $$\begin{array}{rl} g_{x}^{\mathrm{ss}} & =-\Delta (R_{2}+\text{i}\omega_{I}{)}^{-1}g_{y}^{\mathrm{ss}}-\frac{1}{4}A_{1}(R_{2}+\text{i}\omega_{I}{)}^{-1}s_{y}^{\mathrm{ss}},\\ g_{z}^{\mathrm{ss}} & =\omega_{1}(R_{1}+\text{i}\omega_{I}{)}^{-1}g_{y}^{\mathrm{ss}}-\text{i}\frac{1}{4}A_{1}(R_{1}+\text{i}\omega_{I}{)}^{-1}i_{z}^{\mathrm{ss}}. \end{array}$$

Substituting this $g_{z}^{\mathrm{ss}}$ into Eq. ([Disp-formula Ch1.E23]), we obtain

28 $$\begin{array}{rl} {\dot{i}}_{z}{|}_{\mathrm{coh}}^{\mathrm{ss}} & =-\omega_{1}\text{Re}\{\text{i}A_{1}^{*}(R_{1}+\text{i}\omega_{I}{)}^{-1}g_{y}^{\mathrm{ss}}\}\\ & \;-\delta^{2}\text{Re}\{(R_{1}+\text{i}\omega_{I}{)}^{-1}\}i_{z}^{\mathrm{ss}}. \end{array}$$

The first term on the right-hand side of Eq. ([Disp-formula Ch1.E28]) vanishes when $\omega_{1}=0$ . In contrast, the term in the second line is independent of $\omega_{1}$ and thus also contributes in the absence of mw excitation. We thus identify this second term with the thermal relaxation rate

29 $$R_{1I}^{A}=\delta^{2}\text{Re}\{(R_{1}+\text{i}\omega_{I}{)}^{-1}\}.$$

Since we are not interested in this rate, the second summand in Eq. ([Disp-formula Ch1.E28]) can be dropped at this stage. The rate constants $v_{+}$ and $pv_{-}$ will thus be identified using only the first line in Eq. ([Disp-formula Ch1.E28]):

30 $$\omega_{1}\text{Re}\{\text{i}A_{1}^{*}(R_{1}+\text{i}\omega_{I}{)}^{-1}g_{y}^{\mathrm{ss}}\}=v_{+}i_{z}^{\mathrm{ss}}+pv_{-}s_{z}^{\mathrm{eq}}.$$

Substituting $g_{x}^{\mathrm{ss}}$ and $g_{z}^{\mathrm{ss}}$ from Eq. ([Disp-formula Ch1.E27]) into the middle equality of Eq. ([Disp-formula Ch1.E22]) and using the electronic steady state, we find

31 $$\begin{array}{rl} g_{y}^{\mathrm{ss}} & =\frac{1}{4}A_{1}\omega_{1}\Delta P_{0}^{-1}[R_{2}^{-1}+(R_{2}+\text{i}\omega_{I}{)}^{-1}]P^{-1}\;s_{z}^{\mathrm{eq}}\\ & \;+\text{i}\frac{1}{4}A_{1}\omega_{1}(R_{1}+\text{i}\omega_{I}{)}^{-1}P^{-1}\;i_{z}^{\mathrm{ss}}, \end{array}$$

where

32 $$P=R_{2}+\text{i}\omega_{I}+\omega_{1}^{2}(R_{1}+\text{i}\omega_{I}{)}^{-1}+\Delta^{2}(R_{2}+\text{i}\omega_{I}{)}^{-1}$$

generalizes Eq. ([Disp-formula Ch1.E14]) such that $P_{0}=P(\omega_{I}=0)$ . Finally, using this $g_{y}^{\mathrm{ss}}$ in Eq. ([Disp-formula Ch1.E30]), we obtain

33 $$v_{+}=-\delta^{2}\omega_{1}^{2}\;\text{Re}\left\{\frac{(R_{1}+\text{i}\omega_{I}{)}^{-2}}{R_{2}+\text{i}\omega_{I}+\frac{\omega_{1}^{2}}{R_{1}+\text{i}\omega_{I}}+\frac{\Delta^{2}}{R_{2}+\text{i}\omega_{I}}}\right\}$$

and

34 $$\begin{array}{rl} pv_{-} & =-\delta^{2}\omega_{1}^{2}\frac{\Delta }{R_{2}+\omega_{1}^{2}T_{1}+\Delta^{2}T_{2}}\\ & \times \text{Im}\left\{\frac{[R_{2}^{-1}+(R_{2}+\text{i}\omega_{I}{)}^{-1}](R_{1}+\text{i}\omega_{I}{)}^{-1}}{R_{2}+\text{i}\omega_{I}+\frac{\omega_{1}^{2}}{R_{1}+\text{i}\omega_{I}}+\frac{\Delta^{2}}{R_{2}+\text{i}\omega_{I}}}\right\}. \end{array}$$

In these expressions we have written down the combinations P and $P_{0}$ explicitly in order to show in closed form how $v_{+}$ and $pv_{-}$ depend on all the parameters. For example, we immediately see that $pv_{-}$ is odd in the offset Δ , while $v_{+}$ is even. Because the SE-DNP enhancement is proportional to the ratio of these two rates (Eq. [Disp-formula Ch1.E5]), it has the characteristic odd (i.e., antisymmetric) dependence on the offset from the electronic resonance.

When generalizing the SE spin dynamics to g -tensor anisotropy, we will write the dynamical equations (Eq. [Disp-formula Ch1.E21]) as

35 $$\vec{\dot{g}}(t)=-B\vec{g}(t)-\frac{1}{4}A_{1}G\vec{s}(t)-\text{i}\frac{1}{4}A_{1}\hat{k}i_{z}(t)$$

with

36 $$\vec{g}(t)=\left[\begin{array}{c} g_{x}(t)\\ g_{y}(t)\\ g_{z}(t) \end{array}\right],\;G=\left[\begin{array}{ccc} 0 & 1 & 0\\ -1 & 0 & 0\\ 0 & 0 & 0 \end{array}\right]=\frac{\partial B}{\partial \Delta }.$$

### 3.3 Solid effect in liquids

The modulation of the dipolar interaction by translational diffusion was described in [Bibr bib1.bibx45] on the level of the spectral density of the motional model, which was denoted by $J_{11}(s)$ since this is the Laplace transform of the autocorrelation function of the dipolar interaction $A_{1}$ (hence the double subscript of J ). As an example, the spectral density of the FFHS model of translational diffusion is [Bibr bib1.bibx6]

37 $$J_{11}^{\mathrm{ffhs}}(s)=\langle \delta^{2}\rangle \;\tau \frac{(s\tau {)}^{\frac{1}{2}}+4}{(s\tau {)}^{\frac{3}{2}}+4(s\tau )+9(s\tau {)}^{\frac{1}{2}}+9}.$$

Here, the parameter

38 $$\tau =b^{2}/D_{\mathrm{trans}}$$

is the diffusive timescale of the model, which depends on the contact distance of the electronic and nuclear spins, b , and on the coefficient of their relative translational diffusion, $D_{\mathrm{trans}}$ , and

39 $$\langle \delta^{2}\rangle =D_{\mathrm{dip}}^{2}\frac{6\pi }{5}\frac{N}{3b^{3}}$$

is the average of the dipolar interaction strength $\delta^{2}$ over the sample volume times the concentration of the electronic spins, N .

It is convenient to write $J_{11}$ , which has units of angular frequency, as

40 $$J_{11}(s)=\langle \delta^{2}\rangle \;j_{11}(s),$$

where $j_{11}(s)$ has units of time. This factorization confines the effect of the parameters N and b and the constant $D_{\mathrm{dip}}$ to the scaling factor $\langle \delta^{2}\rangle$ . The factor $j_{11}(s)$ then fully accounts for the line shape of the SE-DNP spectrum, which results from the interplay between the offset frequency and the timescale of the translational motion.

According to [Bibr bib1.bibx45], the modification from solids to liquids amounts to replacing the matrix $B^{-1}$ in Eq. ([Disp-formula Ch1.E25]) with the matrix

41 $$Q=j_{11}(B)$$

and also replacing $\delta^{2}$ with $\langle \delta^{2}\rangle$ . The desired SE rate constants in liquids are thus

42 $$\begin{array}{rl} R_{1I}^{A} & =\langle \delta^{2}\rangle \;\text{Re}\{[Q_{\omega_{1}=0}{]}_{zz}\},\\ v_{+} & =\langle \delta^{2}\rangle \;\text{Re}\{[Q{]}_{zz}\}-R_{1I}^{A},\\ pv_{-} & =-\langle \delta^{2}\rangle \;\omega_{1}P_{0}^{-1}\text{Im}\{[Q{]}_{zx}+\Delta T_{2}[Q{]}_{zy}\}. \end{array}$$

We now clarify the meaning of Eq. ([Disp-formula Ch1.E41]). Following the definition of the function of a matrix, one should first solve the eigenvalue problem of B , i.e., BU=UΛ , where the diagonal matrix $\Lambda =\text{diag}(\lambda_{1},\lambda_{2},\lambda_{3})$ contains the three eigenvalues and the columns of U contain the corresponding (right) eigenvectors. Then one should evaluate the spectral density at the three eigenvalues: $\ell_{n}=j_{11}(\lambda_{n})$ . Finally, one should form the diagonal matrix $L=\text{diag}(\ell_{1},\ell_{2},\ell_{3})$ and calculate $Q=ULU^{-1}$ . Comparing this expression of Q with $B^{-1}=U\Lambda U^{-1}$ , we see that, in the transition from solids to liquids, where $B^{-1}$ is replaced with Q , we essentially “process” the eigenvalues of B with the spectral density function $j_{11}$ . This step prevents us from eliminating the variables $g_{x,z}^{\mathrm{ss}}$ in the way we did previously for solids (Sect. [Sec Ch1.S3.SS2]). Because of that, the rate constants in liquids (Eq. [Disp-formula Ch1.E42]) need to be calculated numerically.

Nonetheless, it is still possible to simplify the expression for $R_{1I}^{A}$ , since when $\omega_{1}=0$ , the zz component of B is decoupled from the rest of the matrix. One then finds

43 $$R_{1I}^{A}=\langle \delta^{2}\rangle \;\text{Re}\{j_{11}(R_{1}+\text{i}\omega_{I})\}.$$

Clearly, the time dependence of the dipolar interaction modifies all rate constants, including $R_{1I}^{A}$ (cf. Eq. [Disp-formula Ch1.E29]).

## 4 Slow-motional EPR and DNP spectra for an anisotropic g tensor

In this section we show how to account for g -tensor anisotropies when the tumbling of the radical is slow. Because our description of SE is built around the Bloch equations [Bibr bib1.bibx44], we first adapt the treatment of isotropic rotational diffusion of [Bibr bib1.bibx14] to our needs (Sect. [Sec Ch1.S4.SS1], [Sec Ch1.S4.SS2] and [Sec Ch1.S4.SS3]) and then generalize it to SE-DNP (Sect. [Sec Ch1.S4.SS4] and [Sec Ch1.S4.SS5]). If needed, further generalization to anisotropic diffusion and an orienting potential can be carried out analogously, following the mathematical treatment of the MOMD and SRLS models for slow-motional EPR [Bibr bib1.bibx33].

### 4.1 Stochastic Liouville equation for isotropic tumbling

Following [Bibr bib1.bibx14], we account for the effect of tumbling on the EPR spectrum using the SLE formalism [Bibr bib1.bibx4]. We describe the rotational state of the radical statistically with the probability density P(Ω,t) , which quantifies the likelihood that at time t the molecular system of coordinates in which the g tensor is diagonal will have orientation Ω with respect to the laboratory system of axes defined by the magnetic fields $B_{0}$ and $B_{1}$ . In the case of isotropic rotation, this probability evolves with the Fokker–Planck equation

44 $$\frac{\partial }{\partial t}p(\Omega ,t)=D_{\mathrm{rot}}\nabla_{\Omega }^{2}\;p(\Omega ,t),$$

where $D_{\mathrm{rot}}$ is the rotational diffusion constant of the radical and the Laplace differential operator $\nabla_{\Omega }^{2}$ acts on the orientation variable Ω . The operator

45 $$K_{\Omega }=-D_{\mathrm{rot}}\nabla_{\Omega }^{2}$$

satisfies the following eigenvalue problem:

46 $$K_{\Omega }D_{mn}^{\ell }(\Omega )=D_{\mathrm{rot}}\ell (\ell +1)D_{mn}^{\ell }(\Omega ),$$

where the eigenfunctions $D_{mn}^{\ell }(\Omega )$ are the Wigner rotation matrix elements, which are orthogonal to each other:

47 $$\int D_{MN}^{L*}(\Omega )D_{mn}^{\ell }(\Omega )\;\text{d}\Omega =\frac{8\pi^{2}}{2L+1}\delta_{L\ell }\delta_{Mm}\delta_{Nn}.$$

From Eq. ([Disp-formula Ch1.E46]) it is clear that the time derivative on the left-hand side of Eq. ([Disp-formula Ch1.E44]) vanishes for the equilibrium probability

48 $$p^{\mathrm{eq}}(\Omega )=\frac{1}{8\pi^{2}}=\frac{1}{8\pi^{2}}D_{00}^{0}(\Omega ).$$

In the presence of g -tensor anisotropy, the electronic Larmor frequency depends on the orientation Ω of the radical as follows:We follow [Bibr bib1.bibx14] and consider the effect of the g -tensor anisotropy only on the secular terms in the electronic spin Hamiltonian, i.e., those proportional to the spin operator $S_{z}$ . The response of the non-secular terms to the g anisotropy is neglected.

49 $$\omega (\Omega )=\omega_{0}+\gamma_{0}^{2}D_{00}^{2}(\Omega )+\gamma_{2}^{2}[D_{-20}^{2}(\Omega )+D_{20}^{2}(\Omega )],$$

with the angular frequencies [Bibr bib1.bibx14]

50 $$\begin{array}{rl} \gamma_{0}^{2} & =\frac{2}{3}\left[g_{zz}-\frac{1}{2}(g_{xx}+g_{yy})\right]\;\mu_{B}B_{0}/\hbar ,\\ \gamma_{2}^{2} & =\frac{1}{\sqrt{6}}(g_{xx}-g_{yy})\;\mu_{B}B_{0}/\hbar . \end{array}$$

These are formed from the components $g_{xx}$ , $g_{yy}$ and $g_{zz}$ of the g tensor in the molecular frame. In Eq. ([Disp-formula Ch1.E49]), the first index in the subscripts of the Wigner rotation matrix elements refers to the molecular system of axes, while the second index refers to the laboratory system. The second indices are zero here because we only consider the secular terms, which are proportional to $S_{z}$ .

Since the electronic Larmor frequency depends on Ω , the offset frequency Δ also becomes a function of the molecular orientation. As an example, for a fixed Ω , the Bloch equations (Eq. [Disp-formula Ch1.E17]) should be modified as

51 $$\vec{\dot{s}}(t)=-[B_{0}+F(\Omega )]\vec{s}(t)+R_{1}\hat{k}s_{z}^{\mathrm{eq}},$$

where the orientation dependence is confined to the 3×3 matrix

52 $$F(\Omega )=\left\{\gamma_{0}^{2}D_{00}^{2}(\Omega )+\gamma_{2}^{2}[D_{-20}^{2}(\Omega )+D_{20}^{2}(\Omega )]\right\}G.$$

(The matrix G was introduced in Eq. [Disp-formula Ch1.E36].) It should be stressed, however, that Eq. ([Disp-formula Ch1.E51]) is not a legitimate equation of motion, as it does not account for the dynamics of the orientation Ω .

The SLE formalism remedies this deficiency by introducing the orientation-conditioned averages $\vec{s}(\Omega ,t)$ , whose spatial part evolves according to the Bloch equations (Eq. [Disp-formula Ch1.E51]) and whose Ω dependence evolves according to the diffusion equation (Eq. [Disp-formula Ch1.E44]):

53 $$\begin{array}{rl} & \frac{\partial }{\partial t}\vec{s}(\Omega ,t)=-(K_{\Omega }⊗E+E_{\Omega }⊗B_{0})\;\vec{s}(\Omega ,t)\\ & \;-E_{\Omega }⊗F(\Omega )\;\vec{s}(\Omega ,t)+R_{1}\hat{k}s_{z}^{\mathrm{eq}}p^{\mathrm{eq}}(\Omega ). \end{array}$$

Here E is the 3×3 identity matrix in 3D space and $E_{\Omega }$ is the identity operator in the same abstract space as $K_{\Omega }$ . The outer product ⊗ is needed to create a combined operator that acts simultaneously in both of these spaces.

Since the functions $D_{mn}^{\ell }(\Omega )$ form a complete set, we expand $\vec{s}(\Omega ,t)$ as follows:

54 $$\vec{s}(\Omega ,t)=\frac{1}{8\pi^{2}}\sum_{\ell =0}^{\infty }\sum_{m=-\ell }^{\ell }\sum_{n=-\ell }^{\ell }D_{mn}^{\ell }(\Omega )\;{\vec{s}}_{mn}^{\ell }(t).$$

The coefficients ${\vec{s}}_{mn}^{\ell }$ , which contain the time dependence, can be obtained from $\vec{s}(\Omega ,t)$ using the orthogonality of $D_{mn}^{\ell }(\Omega )$ (Eq. [Disp-formula Ch1.E47]):

55 $${\vec{s}}_{MN}^{L}(t)=(2L+1)\int D_{MN}^{L*}(\Omega )\;\vec{s}(\Omega ,t)\;\text{d}\Omega .$$

Ultimately, the only property that we care about is the integral of the SLE variable $\vec{s}(\Omega ,t)$ over all the orientations:

56 $$\int \vec{s}(\Omega ,t)\;\text{d}\Omega =\int D_{00}^{0}(\Omega )\vec{s}(\Omega ,t)\;\text{d}\Omega ={\vec{s}}_{00}^{0}(t).$$

In that sense, the (vector) coefficient ${\vec{s}}_{00}^{0}(t)$ is the main object of interest, while all the other coefficients ${\vec{s}}_{mn}^{\ell }(t)$ play an auxiliary, bookkeeping role.

Substituting $\vec{s}(\Omega ,t)$ from Eq. ([Disp-formula Ch1.E54]) into Eq. ([Disp-formula Ch1.E53]), multiplying both sides by $D_{MN}^{L*}(\Omega )$ and integrating over Ω , we get

57 $$\begin{array}{rl} & {\vec{\dot{s}}}_{MN}^{L}(t)=R_{1}\hat{k}s_{z}^{\mathrm{eq}}\delta_{L0}\delta_{M0}\delta_{N0}\\ & -[D_{\mathrm{rot}}L(L+1)+B_{0}]{\vec{s}}_{MN}^{L}(t)\\ & -\sum_{\ell mn}\left(\frac{2L+1}{8\pi^{2}}\int D_{MN}^{L*}(\Omega )D_{mn}^{\ell }(\Omega )F(\Omega )\;\text{d}\Omega \right){\vec{s}}_{mn}^{\ell }(t). \end{array}$$

Clearly, the terms proportional to $K_{\Omega }$ and $B_{0}$ in Eq. ([Disp-formula Ch1.E53]) do not mix coefficients ${\vec{s}}_{MN}^{L}$ with different values of L , M and N . In other words, these two operators are diagonal in the selected representation. The term proportional to F(Ω) , on the other hand, mixes coefficients with different L and M (but not N , as we discuss below).

The integral in the last line of Eq. ([Disp-formula Ch1.E57]) contains the product of three Wigner rotation matrix elements. These can be expressed in terms of the Clebsch–Gordan coefficients $C_{\ell_{1}m_{1}\ell_{2}m_{2}}^{LM}$ . Specifically, for the $D_{K0}^{2}(\Omega )$ in Eq. ([Disp-formula Ch1.E52]), we have

58 $$\frac{2L+1}{8\pi^{2}}\int D_{MN}^{L*}(\Omega )D_{K0}^{2}(\Omega )D_{mn}^{\ell }(\Omega )\;\text{d}\Omega =C_{2K\ell m}^{LM}C_{20\ell n}^{LN},$$

which leads to

59 $$\begin{array}{rl} & {\vec{\dot{s}}}_{MN}^{L}(t)=R_{1}\hat{k}s_{z}^{\mathrm{eq}}\delta_{L0}\delta_{M0}\delta_{N0}\\ & -[D_{\mathrm{rot}}L(L+1)+B_{0}]{\vec{s}}_{MN}^{L}(t)\\ & -\sum_{\ell mn}[\gamma_{0}^{2}C_{20\ell m}^{LM}+\gamma_{2}^{2}(C_{2-2\ell m}^{LM}+C_{22\ell m}^{LM})]C_{20\ell n}^{LN}G{\vec{s}}_{mn}^{\ell }(t). \end{array}$$

In Eq. ([Disp-formula Ch1.E59]), the sum over ℓ mixes only expansion coefficients with ℓ=L,L±2 [Bibr bib1.bibx14] because all three Wigner rotation matrix elements in F have L=2 (Eq. [Disp-formula Ch1.E52]). Since we need ${\vec{s}}_{00}^{0}$ at the end, it is sufficient to consider only coefficients with even values of ℓ . Furthermore, as the Wigner rotation matrix elements in F have M=0±2 and N=0 , the sum over m mixes only coefficients whose values m are either equal to M or differ from it by two units, while the sum over n does not mix any coefficients with n different from N . These considerations imply that the triple sum in Eq. ([Disp-formula Ch1.E59]) will only go over ${\vec{s}}_{m0}^{\ell }$ with even ℓ and m . Finally, because the Wigner rotation matrix elements with M=2 and M=-2 appear in a symmetrical way in F , it becomes possible to work with the symmetrized coefficients [Bibr bib1.bibx14]

60 $${\vec{s}}^{LM}=\frac{1}{2}({\vec{s}}_{-M0}^{L}+{\vec{s}}_{M0}^{L}),$$

thus restricting M to non-negative values ( 0≤M≤L ). The lowest-order coefficients that are coupled by the SLE dynamics are thus ${\vec{s}}^{00}$ , ${\vec{s}}^{20}$ , ${\vec{s}}^{22}$ , ${\vec{s}}^{40}$ , ${\vec{s}}^{42}$ , ${\vec{s}}^{44}$ , ${\vec{s}}^{60}$ , etc.

### 4.2 Matrix representation of the SLE dynamics

While the above considerations greatly reduce the needed coefficients, there are still an infinite number left. In any practical work, this infinite set is truncated by selecting a maximum value of L to account for and setting to zero the coefficients with $L>L_{\max}$ . Since the total number of even L such that $L\leq L_{\max}$ is $n_{L}=L_{\max}/2+1$ , the total number of remaining coefficients ${\vec{s}}^{LM}$ is $n_{\mathrm{tot}}=n_{L}(n_{L}+1)/2=L_{\max}^{2}/8+3L_{\max}/4+1$ . For the smallest non-trivial choice of $L_{\max}=2$ , $n_{\mathrm{tot}}=3$ (with ${\vec{s}}^{00}$ , ${\vec{s}}^{20}$ and ${\vec{s}}^{22}$ ). The number of coefficients increases quadratically with $L_{\max}$ (e.g., $n_{\mathrm{tot}}=15,28,45$ for $L_{\max}=8,12,16$ , respectively).

To compactly write down how these coefficients are mixed by the SLE dynamics, we introduce the following abstract vectors with $n_{\mathrm{tot}}$ elements:

61 $$1^{00}=\left[\begin{array}{c} 1\\ 0\\ 0\\ \vdots \end{array}\right],\;s_{i}(t)=\left[\begin{array}{c} s_{i}^{00}(t)\\ s_{i}^{20}(t)\\ s_{i}^{22}(t)\\ \vdots \end{array}\right]\;(i=x,y,z),$$

where the former is needed for the first term on the right-hand side of Eq. ([Disp-formula Ch1.E59]). The SLE dynamics then becomes

62 $$\left[\begin{array}{c} {\dot{s}}_{x}(t)\\ {\dot{s}}_{y}(t)\\ {\dot{s}}_{z}(t) \end{array}\right]=-B_{0}\left[\begin{array}{c} s_{x}(t)\\ s_{y}(t)\\ s_{z}(t) \end{array}\right]+R_{1}\left[\begin{array}{c} 0\\ 0\\ 1^{00} \end{array}\right]s_{z}^{\mathrm{eq}},$$

where

63 $$B_{0}=\left[\begin{array}{ccc} R_{2} & \Delta & 0\\ -\Delta & R_{2} & \omega_{1}E\\ 0 & -\omega_{1}E & R_{1} \end{array}\right]$$

is a $3n_{\mathrm{tot}}\times 3n_{\mathrm{tot}}$ matrix, and E , $R_{1}$ , $R_{2}$ and Δ are $n_{\mathrm{tot}}\times n_{\mathrm{tot}}$ matrices.

The first three of these sub-matrices are purely diagonal: E is the identity matrix and

64 $$R_{1,2}=R_{1,2}E+D_{\mathrm{rot}}C_{D},$$

with the diagonal elements of $C_{D}$ being equal to L(L+1) . For the simplest case of $L_{\max}=2$ with only three coefficients ( ${\vec{s}}^{00}$ , ${\vec{s}}^{20}$ and ${\vec{s}}^{22}$ ),

65 $$E=\left[\begin{array}{ccc} 1 & & \\ & 1 & \\ & & 1 \end{array}\right],\;C_{D}=\left[\begin{array}{ccc} 0 & & \\ & 6 & \\ & & 6 \end{array}\right].$$

In Eq. ([Disp-formula Ch1.E63]), the diagonal matrices $R_{1,2}$ and E , which originate from the second line of Eq. ([Disp-formula Ch1.E59]), do not mix coefficients with different L and M . Only the sub-matrix Δ , which is of the form

66 $$\Delta =\Delta E+\gamma_{0}^{2}C_{0}+\gamma_{2}^{2}C_{2},$$

mixes coefficients of different orders. In fact, the mixing is due to the matrices $C_{0,2}$ , which modify the frequency offset Δ in proportion to the g -tensor anisotropies $\gamma_{0}^{2}$ and $\gamma_{2}^{2}$ . For $L_{\max}=2$ ,

67 $$C_{0}=\left[\begin{array}{ccc} 0 & \frac{1}{5} & 0\\ 1 & \frac{2}{7} & 0\\ 0 & 0 & -\frac{2}{7} \end{array}\right],\;C_{2}=\left[\begin{array}{ccc} 0 & 0 & \frac{1}{5}\times 2\\ 0 & 0 & -\frac{2}{7}\times 2\\ 1 & -\frac{2}{7} & 0 \end{array}\right].$$

(The factors of 2 in the last column of $C_{2}$ arise from the fact that coefficients with M=0 pose an exception to the symmetrization in Eq. [Disp-formula Ch1.E60].) The matrix elements of these two matrices in the most general case are

68 $$\begin{array}{rl} {}[C_{0}{]}_{LM,\ell m} & =C_{20\ell m}^{LM}C_{20\ell 0}^{L0},\\ {}[C_{2}{]}_{LM,\ell m} & =(C_{2-2\ell m}^{LM}+C_{22\ell m}^{LM}+\delta_{M0}C_{22\ell m}^{LM})C_{20\ell 0}^{L0}, \end{array}$$

where the summand proportional to $\delta_{M0}$ in the second line accounts for the factor of 2 that is needed by the coefficients ${\vec{s}}^{L0}$ .

Selecting $L_{\max}=0$ in the above formalism amounts to retaining only the (3D vector) coefficient ${\vec{s}}^{00}$ . Then the matrix $B_{0}$ in Eq. ([Disp-formula Ch1.E63]) reduces to $B_{0}$ , and Eq. ([Disp-formula Ch1.E62]) reduces to the classical Bloch equations for a homogeneous line. For $L_{\max}>0$ , the diagonal matrices $R_{1}$ and $R_{2}$ cause the coefficients $s_{z}^{LM}$ and $s_{x,y}^{LM}$ , respectively, to decay exponentially, with those with larger L being suppressed more strongly by the tumbling. Analogously to the Bloch equations, the mw excitation mixes the y and z components of ${\vec{s}}^{LM}$ without mixing their LM dependence. The latter is mixed only by the offset matrix Δ , as elaborated above.

By building the SLE dynamics on top of the classical Bloch equations, we have arrived at a rather intuitive picture of how the g -tensor anisotropy is incorporated into the spin dynamics. Specifically, every element of the Bloch matrix $B_{0}$ (Eq. [Disp-formula Ch1.E19] with $\omega_{I}=0$ ) is replaced with a matrix in the space of LM indices (Eq. [Disp-formula Ch1.E63]). In this replacement, all the elements except the frequency offset become diagonal matrices in the LM space, with the mixing in this space being entirely due to the offset. Since we describe the solid effect by two coupled Bloch equations, this intuition about the effect of g -tensor anisotropy on the spin dynamics will be helpful when adapting the approach to SE-DNP in Sect. 4.4 and 4.5.

### 4.3 EPR spectrum and saturation

The cw-EPR spectrum and the electronic saturation factor under g -tensor anisotropy are obtained from the steady state of Eq. ([Disp-formula Ch1.E62]),

69 $$B_{0}\left[\begin{array}{c} s_{x}^{\mathrm{ss}}\\ s_{y}^{\mathrm{ss}}\\ s_{z}^{\mathrm{ss}} \end{array}\right]=R_{1}\left[\begin{array}{c} 0\\ 0\\ 1^{00} \end{array}\right]s_{z}^{\mathrm{eq}},$$

which can be solved by inverting the $3n_{\mathrm{tot}}\times 3n_{\mathrm{tot}}$ matrix $B_{0}$ numerically. However, it is also possible to solve Eq. ([Disp-formula Ch1.E69]) by inverting a single matrix with dimensions that are 3 times smaller (i.e., $n_{\mathrm{tot}}\times n_{\mathrm{tot}}$ ), as we show next.

First, taking advantage of the zeros in $B_{0}$ (Eq. [Disp-formula Ch1.E63]), we express $s_{x}^{\mathrm{ss}}$ and $s_{z}^{\mathrm{ss}}$ in terms of $s_{y}^{\mathrm{ss}}$ :

70 $$\begin{array}{rl} s_{x}^{\mathrm{ss}} & =-R_{2}^{-1}\Delta \;s_{y}^{\mathrm{ss}},\\ s_{z}^{\mathrm{ss}} & =\omega_{1}R_{1}^{-1}s_{y}^{\mathrm{ss}}+R_{1}R_{1}^{-1}1^{00}s_{z}^{\mathrm{eq}}. \end{array}$$

Because only the first element of $1^{00}$ is non-zero and the diagonal matrix $R_{1}$ does not mix coefficients with different values of LM , the second equality in Eq. ([Disp-formula Ch1.E70]) becomes

71 $$s_{z}^{\mathrm{ss}}=\omega_{1}R_{1}^{-1}s_{y}^{\mathrm{ss}}+1^{00}s_{z}^{\mathrm{eq}}.$$

For the 00th (i.e., first) element of $s_{z}^{\mathrm{ss}}$ , we thus have $s_{z}^{00}=\omega_{1}T_{1}s_{y}^{00}+s_{z}^{\mathrm{eq}}$ , which is identical to the second equality in Eq. ([Disp-formula Ch1.E12]). We thus conclude that the proportionality between the electronic saturation factor and the in-phase EPR line shape (Eq. [Disp-formula Ch1.E16]) is not limited to a homogenous line but also applies under g -tensor anisotropy, at least in the case of isotropic rotational diffusion.

Second, from the middle row of the matrix $B_{0}$ (Eq. [Disp-formula Ch1.E63]) and after substituting $s_{x}^{\mathrm{ss}}$ and $s_{z}^{\mathrm{ss}}$ from Eq. ([Disp-formula Ch1.E70]), we find

72 $$s_{y}^{\mathrm{ss}}=-\omega_{1}P_{0}^{-1}1^{00}s_{z}^{\mathrm{eq}},$$

where we have introduced the $n_{\mathrm{tot}}\times n_{\mathrm{tot}}$ matrix

73 $$P=(R_{2}+\text{i}\omega_{I})+\omega_{1}^{2}(R_{1}+\text{i}\omega_{I}{)}^{-1}+\Delta (R_{2}+\text{i}\omega_{I}{)}^{-1}\Delta$$

and $P_{0}=P(\omega_{I}=0)$ . The matrix $P_{0}$ generalizes $P_{0}$ (Eq. [Disp-formula Ch1.E14]), and Eq. ([Disp-formula Ch1.E72]) generalizes Eq. ([Disp-formula Ch1.E13]) to the case of g -tensor anisotropy.

From the 00th components of $s_{y}^{\mathrm{ss}}$ and $s_{x}^{\mathrm{ss}}$ , we find

74 $$\mathrm{abs}=-\omega_{1}[P_{0}^{-1}{]}_{11},\;\mathrm{dsp}=\omega_{1}T_{2}[\Delta P_{0}^{-1}{]}_{11},$$

where we used the fact that $R_{2}$ is a diagonal matrix. These expressions generalize Eq. ([Disp-formula Ch1.E15]) to the case of g -tensor anisotropy. The corresponding saturation factor as a function of the offset is then (from Eqs. [Disp-formula Ch1.E16] and [Disp-formula Ch1.E74])

75 $$s(\Delta )=\omega_{1}^{2}T_{1}[P_{0}^{-1}(\Delta ){]}_{11}.$$

As claimed, to solve for the steady state numerically, we need to invert the matrix $P_{0}$ , whose dimensions are 3 times smaller than those of $B_{0}$ . (The two matrix inversions needed to calculate $P_{0}$ itself involve the diagonal matrices $R_{1,2}$ .)

The cw-EPR spectrum in derivative mode can be calculated from the derivative of $P_{0}$ with respect to the (scalar) frequency offset Δ :

76 $$\frac{\partial P_{0}}{\partial \Delta }=R_{2}^{-1}\Delta +\Delta R_{2}^{-1}.$$

The in-phase and out-of-phase derivative spectra are then obtained from the first (i.e., 00th) components of the vectors

77 $$\begin{array}{rl} \frac{\partial s_{y}}{\partial \Delta } & =\omega_{1}P_{0}^{-1}(R_{2}^{-1}\Delta +\Delta R_{2}^{-1})P_{0}^{-1}1^{00}s_{z}^{\mathrm{eq}},\\ \frac{\partial s_{x}}{\partial \Delta } & =-R_{2}^{-1}(s_{y}+\Delta \frac{\partial s_{y}}{\partial \Delta }). \end{array}$$

These expressions are used in Sect. [Sec Ch1.S5] to fit the experimental EPR spectra from Fig. [Fig Ch1.F1].

**Figure 2 Ch1.F2:**
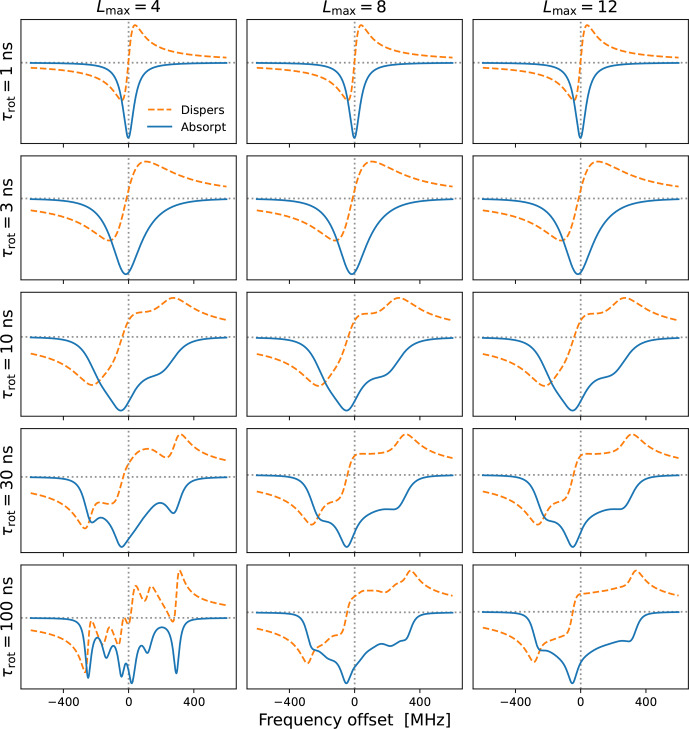
Cw-EPR spectra for g=diag(2.00755,2.00555,2.0023) and different tumbling times $\tau_{\mathrm{rot}}$ . A larger $L_{\max}$ is necessary for slower tumbling. The needed $L_{\max}$ also depends on the anisotropies, which are $\left(\gamma_{0},\gamma_{2})=(-373,107\right)$ MHz for $B_{0}=9.403$ T. Other simulation parameters were $B_{1}=0.02$ G, $T_{1}=100$ ns and $T_{2}=20$ ns. Note that these are the values of $T_{1}$ and $T_{2}$ that are introduced by hand. The rotational tumbling itself further shortens the actual spin–spin relaxation time $T_{2}$ .

In Fig. [Fig Ch1.F2] we show examples of (integral) EPR spectra calculated using the presented approach for different tumbling times $\tau_{\mathrm{rot}}$ . The different columns in the figure correspond to different choices of $L_{\max}$ . The g -tensor values used in the simulations are characteristic of nitroxide spin labels. We also selected a small mw magnetic field ( $B_{1}=0.02$ G) to mimic the low-power conditions typical of cw-EPR. The main message of this figure is that slower tumbling requires larger $L_{\max}$ . At the same time, we see that $L_{\max}=8$ is already good enough for $\tau_{\mathrm{rot}}\leq 10$ ns, which is the range of rotational timescales of relevance to our experimental data (Sect. [Sec Ch1.S5]). By selecting $L_{\max}=10$ , to be on the safe side, we only need to invert a 21×21 matrix at every frequency offset, which makes the calculation of g -broadened EPR spectra very fast. This allows us to perform an automated search over the various parameters and to fit the experimental cw-EPR spectra in less than a minute.

### 4.4 Solid effect in “solids”

Extending the above treatment to SE-DNP, we combine the spin dynamics in Eq. ([Disp-formula Ch1.E35]) with the rotational dynamics in Eq. ([Disp-formula Ch1.E44]) to form the following SLE:

78 $$\begin{array}{rl} & \frac{\partial }{\partial t}\vec{g}(\Omega ,t)=-(K_{\Omega }⊗E+E_{\Omega }⊗B)\vec{g}(\Omega ,t)\\ & \;-E_{\Omega }⊗F(\Omega )\vec{g}(\Omega ,t)\\ & \;-\frac{1}{4}A_{1}G\vec{s}(\Omega ,t)-\text{i}\frac{1}{4}A_{1}\hat{k}i_{z}(t)p^{\mathrm{eq}}(\Omega ). \end{array}$$

As before, we introduce the expansion

79 $$\vec{g}(\Omega ,t)=\frac{1}{8\pi^{2}}\sum_{\ell =0}^{\infty }\sum_{m=-\ell }^{\ell }D_{m0}^{2}(\Omega )\;{\vec{g}}_{m0}^{\ell }(t),$$

where we have set n=0 from the start and find

80 $$\begin{array}{rl} & {\vec{\dot{g}}}_{M0}^{L}(t)=-[D_{\mathrm{rot}}L(L+1)+B]{\vec{g}}_{M0}^{L}(t)\\ & -\sum_{\ell m}[\gamma_{0}^{2}C_{20\ell m}^{LM}+\gamma_{2}^{2}(C_{2-2\ell m}^{LM}+C_{22\ell m}^{LM})]C_{20\ell n}^{L0}G{\vec{g}}_{m0}^{\ell }(t)\\ & -\frac{1}{4}A_{1}G\;{\vec{s}}_{M0}^{L}(t)-\text{i}\frac{1}{4}A_{1}\hat{k}\;i_{z}(t)\delta_{L0}\delta_{M0}. \end{array}$$

Again, we switch to the symmetrized coefficients

81 $${\vec{g}}^{LM}=\frac{1}{2}({\vec{g}}_{-M0}^{L}+{\vec{g}}_{M0}^{L})$$

and form the following three $n_{\mathrm{tot}}$ -dimensional vectors from the spatial components of the 3D vectors ${\vec{g}}^{LM}$ :

82 $$g_{i}=\left[\begin{array}{c} g_{i}^{00}\\ g_{i}^{20}\\ g_{i}^{22}\\ \vdots \end{array}\right]\;(i=x,y,z).$$

The steady state of the resulting spin dynamics is then

83 $$B\left[\begin{array}{c} g_{x}^{\mathrm{ss}}\\ g_{y}^{\mathrm{ss}}\\ g_{z}^{\mathrm{ss}} \end{array}\right]=-\frac{1}{4}A_{1}\left[\begin{array}{c} s_{y}^{\mathrm{ss}}\\ -s_{x}^{\mathrm{ss}}\\ 0 \end{array}\right]-\text{i}\frac{1}{4}A_{1}\left[\begin{array}{c} 0\\ 0\\ 1^{00} \end{array}\right]i_{z}^{\mathrm{ss}},$$

where

84 $$B=B_{0}+\text{i}\omega_{I}$$

generalizes the matrix $B_{0}$ from Eq. ([Disp-formula Ch1.E63]).

Our goal is to solve for $g_{z}^{\mathrm{ss}}$ , since its 00th component should be used in Eq. ([Disp-formula Ch1.E23]) to calculate the rate constants $pv_{-}$ and $v_{+}$ . After inverting B in Eq. ([Disp-formula Ch1.E83]), we find

85 $$\begin{array}{rl} g_{z}^{\mathrm{ss}} & =-\frac{1}{4}A_{1}[B^{-1}{]}_{zx}s_{y}^{\mathrm{ss}}-\frac{1}{4}A_{1}[B^{-1}{]}_{zy}(-s_{x}^{\mathrm{ss}})\\ & \;-\text{i}\frac{1}{4}A_{1}[B^{-1}{]}_{zz}1^{00}i_{z}^{\mathrm{ss}}. \end{array}$$

Note that now $[B^{-1}{]}_{ij}$ denotes the $n_{\mathrm{tot}}\times n_{\mathrm{tot}}$ sub-matrix of $B^{-1}$ at position ij and not a scalar matrix element. Using $s_{x,y}^{\mathrm{ss}}$ from the previous subsection, we find that the first component of $g_{z}^{\mathrm{ss}}$ is

86 $$\begin{array}{rl} g_{z}^{00} & =\omega_{1}\frac{1}{4}A_{1}[([B^{-1}{]}_{zx}+[B^{-1}{]}_{zy}R_{2}^{-1}\Delta )P_{0}^{-1}{]}_{11}s_{z}^{\mathrm{eq}}\\ & \;-\text{i}\frac{1}{4}A_{1}[[B^{-1}{]}_{zz}{]}_{11}i_{z}^{\mathrm{ss}}. \end{array}$$

Substituting this result into Eq. ([Disp-formula Ch1.E23]), we obtain

87 $$\begin{array}{rl} R_{1I}^{A} & =\delta^{2}\text{Re}\{[[B_{\omega_{1}=0}^{-1}{]}_{zz}{]}_{11}\},\\ v_{+} & =\delta^{2}\text{Re}\{[[B^{-1}{]}_{zz}{]}_{11}\}-R_{1I}^{A},\\ pv_{-} & =-\delta^{2}\omega_{1}\text{Im}\{[([B^{-1}{]}_{zx}+[B^{-1}{]}_{zy}R_{2}^{-1}\Delta )P_{0}^{-1}{]}_{11}\}. \end{array}$$

These expressions, which require the inversion of the $3n_{\mathrm{tot}}\times 3n_{\mathrm{tot}}$ matrix B , are directly generalizable to liquids (Sect. [Sec Ch1.S4.SS5]). In “solids”, it is possible to obtain alternative expressions that require the inversion of a smaller $n_{\mathrm{tot}}\times n_{\mathrm{tot}}$ matrix. To this end, we express $g_{x}^{\mathrm{ss}}$ and $g_{z}^{\mathrm{ss}}$ in terms of $g_{y}^{\mathrm{ss}}$ using the first and last rows of B :

88 $$\begin{array}{rl} g_{x}^{\mathrm{ss}} & =-(R_{2}+\text{i}\omega_{I}{)}^{-1}\Delta g_{y}^{\mathrm{ss}}-\frac{1}{4}A_{1}(R_{2}+\text{i}\omega_{I}{)}^{-1}s_{y}^{\mathrm{ss}},\\ g_{z}^{\mathrm{ss}} & =\omega_{1}(R_{1}+\text{i}\omega_{I}{)}^{-1}g_{y}^{\mathrm{ss}}-\text{i}\frac{1}{4}A_{1}(R_{1}+\text{i}\omega_{I}{)}^{-1}1^{00}i_{z}^{\mathrm{ss}}. \end{array}$$

Substituting the first (i.e., 00th) component of $g_{z}^{\mathrm{ss}}$ into Eq. ([Disp-formula Ch1.E23]), we find

89 $$R_{1I}^{A}=\delta^{2}\text{Re}\{[(R_{1}+\text{i}\omega_{I}{)}^{-1}{]}_{11}\}.$$

Because $R_{1}$ is a diagonal matrix, this result is identical to Eq. ([Disp-formula Ch1.E29]), showing that $R_{1I}^{A}$ is not affected by the anisotropy of the g tensor in the case of isotropic rotation.

Similarly, from the middle part of B , we obtain

90 $$\begin{array}{rl} Pg_{y}^{\mathrm{ss}} & =\frac{1}{4}A_{1}[s_{x}^{\mathrm{ss}}-\Delta (R_{2}+\text{i}\omega_{I}{)}^{-1}s_{y}^{\mathrm{ss}}]\\ & \;+\omega_{1}\text{i}\frac{1}{4}A_{1}(R_{1}+\text{i}\omega_{I}{)}^{-1}1^{00}i_{z}^{\mathrm{ss}}. \end{array}$$

We first observe that $(R_{1}+\text{i}\omega_{I}{)}^{-1}1^{00}=1^{00}(R_{1}+\text{i}\omega_{I}{)}^{-1}$ because $R_{1}$ is diagonal. Then we substitute $s_{x,y}^{\mathrm{ss}}$ from before to get

91 $$\begin{array}{rl} g_{y}^{\mathrm{ss}} & =\omega_{1}\frac{1}{4}A_{1}P^{-1}[R_{2}^{-1}\Delta +\Delta (R_{2}+\text{i}\omega_{I}{)}^{-1}]P_{0}^{-1}1^{00}s_{z}^{\mathrm{eq}}\\ & \;+\omega_{1}\text{i}\frac{1}{4}A_{1}P^{-1}1^{00}(R_{1}+\text{i}\omega_{I}{)}^{-1}i_{z}^{\mathrm{ss}}. \end{array}$$

Finally, substituting the 00th element of $g_{y}^{\mathrm{ss}}$ into Eq. ([Disp-formula Ch1.E30]), we find

92 $$\begin{array}{rl} v_{+} & =-\delta^{2}\omega_{1}^{2}\;\text{Re}\{(R_{1}+\text{i}\omega_{I}{)}^{-2}[P^{-1}{]}_{11}\},\\ pv_{-} & =-\delta^{2}\omega_{1}^{2}\text{Im}\{(R_{1}+\text{i}\omega_{I}{)}^{-1}\\ & \;\times [P^{-1}(R_{2}^{-1}\Delta +\Delta (R_{2}+\text{i}\omega_{I}{)}^{-1})P_{0}^{-1}{]}_{11}\}. \end{array}$$

Observe how these expressions generalize Eqs. ([Disp-formula Ch1.E33]) and ([Disp-formula Ch1.E34]) to the case of g -tensor anisotropy.

**Figure 3 Ch1.F3:**
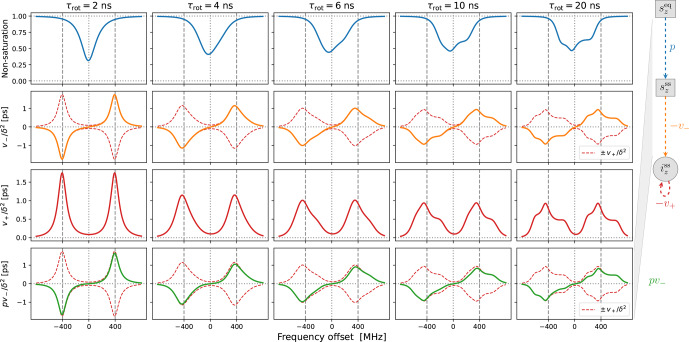
Solid-effect rates $v_{+}$ (red line) and $pv_{-}$ (green line) calculated at high mw power ( $B_{1}=5.5$ G) for static dipolar interaction (i.e., “solid”) and several different rates of rotational tumbling. The factorization of $pv_{-}$ into p (blue line) and $v_{-}$ (orange line) is also shown. All other parameters are as in Fig. [Fig Ch1.F2], and $L_{\max}=10$ . In particular, $T_{1}=100$ ns.

In the last two rows of Fig. [Fig Ch1.F3] we show $v_{+}/\delta^{2}$ and $pv_{-}/\delta^{2}$ , which have units of time. Although the electronic non-saturation factor p and the rate constant $v_{-}$ always appear together as $pv_{-}$ , it is helpful to separate these two factors when rationalizing SE. We show p and $v_{-}/\delta^{2}$ in the first two rows of Fig. [Fig Ch1.F3]. Note that $v_{+}/\delta^{2}$ and $pv_{-}/\delta^{2}$ were calculated directly from Eq. ([Disp-formula Ch1.E92]), whereas $v_{-}/\delta^{2}$ was determined by dividing $pv_{-}/\delta^{2}$ by $p=1-\omega_{1}^{2}T_{1}[P_{0}^{-1}{]}_{11}$ (Eq. [Disp-formula Ch1.E75]).

The columns in Fig. [Fig Ch1.F3] reveal the effect of the g -tensor anisotropy on the different factors relevant to SE. $v_{+}/\delta^{2}$ in the third row of the figure is composed of two SE lines centered at $-\omega_{I}$ and $+\omega_{I}$ . At the fastest tumbling (leftmost column), each of these two lines is symmetric and approximately Lorentzian. When the tumbling slows down, each line broadens and becomes asymmetric. At the slowest tumbling rate (rightmost column), each line resembles a powder EPR spectrum with an anisotropic g tensor. We see that in the regime of slow tumbling the profile of $v_{+}/\delta^{2}$ is no longer symmetric (i.e., even) with respect to the electronic resonance at zero offset frequency.

In the second row of Fig. [Fig Ch1.F3] we show $v_{-}/\delta^{2}$ (orange line), which is also composed of two SE lines centered at $-\omega_{I}$ and $+\omega_{I}$ , with the former flipped with respect to the horizontal axis. For comparison, in the second row we also plotted $v_{+}/\delta^{2}$ and $-v_{+}/\delta^{2}$ (dashed red lines). We see that, for all the tumbling rates, the two SE lines comprising $v_{-}/\delta^{2}$ exactly match their counterparts in $v_{+}/\delta^{2}$ .

The first row of Fig. [Fig Ch1.F3] shows the electronic saturation under g -tensor anisotropy (we actually plot the “non-saturation” p=1-s ). Because of the large $B_{1}$ used in the calculations ( $B_{1}=5.5$ G), appreciable electronic saturation is achieved for all the shown tumbling rates. From the perspective of the solid effect, it is noteworthy that the saturation is more localized to on-resonance conditions when the g -tensor anisotropy is averaged out by the tumbling and spreads to larger off-resonance frequencies when the tumbling slows down. This spread broadens the saturation profile and reduces its maximum. However, in spite of the substantial increase in the spectral width of the saturation when going from $\tau_{\mathrm{rot}}=2$ ns to $\tau_{\mathrm{rot}}=20$ ns, the maximum decreases only moderately, remaining close to 50 % at the slower tumbling rate.

Of course, the amplitude of the saturation profile depends not only on $B_{1}$ , but also on the electronic $T_{1}$ relaxation time. To illustrate this dependence, we recalculated all the curves in Fig. [Fig Ch1.F3] after increasing $T_{1}$ 5-fold to 500 ns. The result, which is shown in Fig. [Fig App1.Ch1.S1.F8], demonstrates larger saturation for all tumbling rates. At the same time, $v_{-}/\delta^{2}$ and $v_{+}/\delta^{2}$ (second and third rows) remain entirely unaffected. This demonstrates that, in our case of a high constant magnetic field, the SE lines do not experience the power broadening that affects the EPR spectrum.

Finally, the last row of Fig. [Fig Ch1.F3] shows $pv_{-}/\delta^{2}$ (solid green line), which equals the product of the first and second rows. From Eq. ([Disp-formula Ch1.E7]), we know that $pv_{-}/\delta^{2}$ basically gives the SE-DNP spectrum up to an overall scaling factor. Since $pv_{-}$ is suppressed by the electronic saturation compared to $v_{-}$ , we see that $pv_{-}/\delta^{2}$ is somewhat reduced at offsets between the canonical SE positions $\pm \omega_{I}$ . Because both the electronic saturation profile and the profile of $v_{-}$ are asymmetric in the slow motional regime where the EPR line exhibits clear g broadening, the line shape of the SE-DNP spectrum (proportional to $pv_{-}$ ) is no longer antisymmetric (i.e., odd) with respect to the electronic resonance. This is most visible for the green line in the lower rightmost corner of Fig. [Fig Ch1.F3].

### 4.5 Solid effect in liquids

In the light of Sect. [Sec Ch1.S3.SS3], the generalization to liquids consists of calculating the matrix

93 $$Q=j_{11}(B)$$

and using it instead of $B^{-1}$ in Eq. ([Disp-formula Ch1.E87]):

94 $$\begin{array}{rl} R_{1I}^{A} & =\langle \delta^{2}\rangle \;\text{Re}\{[[Q_{\omega_{1}=0}{]}_{zz}{]}_{11}\},\\ v_{+} & =\langle \delta^{2}\rangle \;\text{Re}\{[[Q{]}_{zz}{]}_{11}\}-R_{1I}^{A},\\ pv_{-} & =-\langle \delta^{2}\rangle \;\omega_{1}\text{Im}\{[([Q{]}_{zx}+[Q{]}_{zy}R_{2}^{-1}\Delta )P_{0}^{-1}{]}_{11}\}. \end{array}$$

Because the zz sub-block of B is diagonal and does not couple to the rest when $\omega_{1}=0$ , we deduce that

95 $$R_{1I}^{A}=\langle \delta^{2}\rangle \;\text{Re}\{j_{11}(R_{1}+\text{i}\omega_{I})\},$$

which is identical to Eq. ([Disp-formula Ch1.E43]). Thus, as we already observed for “solids”, the expression for $R_{1I}^{A}$ is not affected by the anisotropy of the g tensor and the slow tumbling of the radical, in the case of isotropic rotational diffusion.

In Fig. [Fig Ch1.F4] we show the same properties as in Fig. [Fig Ch1.F3] but now in the presence of translational diffusion treated by the FFHS model with the motional timescale $\tau_{\mathrm{ffhs}}=6$ ns. Several changes compared to “solids” (Fig. [Fig Ch1.F3]) are worth pointing out.

**Figure 4 Ch1.F4:**
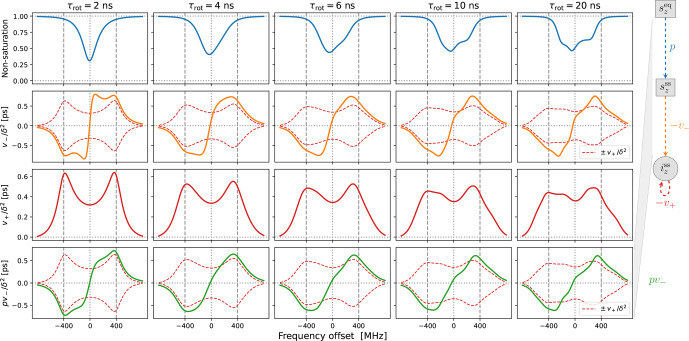
Same as Fig. [Fig Ch1.F3] but for the FFHS model of translational diffusion with $\tau_{\mathrm{ffhs}}=6$ ns.

**Figure 5 Ch1.F5:**
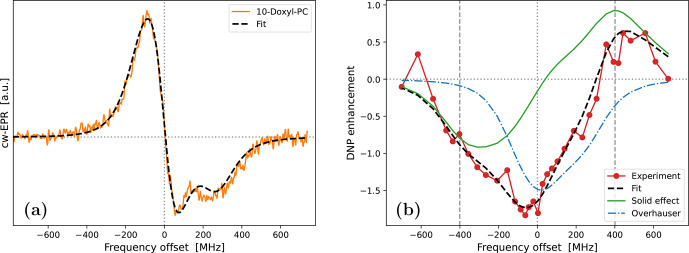
Fits to the experimental cw-EPR spectrum **(a)** and DNP spectrum **(b)** of 10-Doxyl-PC. In both cases, our best fits are shown with dashed black lines. The DNP spectrum in panel **(b)** is calculated by adding the contributions of SE (solid green line) and OE (dotted–dashed blue line), both of which are affected by the g -tensor anisotropy. The fit parameters are given in Table [Table Ch1.T1].

In line with our previous understanding [Bibr bib1.bibx45], the SE lines comprising $v_{+}/\langle \delta^{2}\rangle$ are broadened by the translational motion that modulates the dipolar interaction (red lines in the third row of Fig. [Fig Ch1.F4]). This motional broadening reduces their maximum intensities compared to “solids” (Fig. [Fig Ch1.F3], third row). Previously, in the case of Lorentzian lines, the reduction in intensity in the transition from solids to liquids was dramatic, by more than a factor of 10 ([Bibr bib1.bibx45], Figs. 3, 4 and 5). In contrast, the reduction in the presence of g -tensor broadening is about a factor of 2 (compare the third rows of Figs. [Fig Ch1.F3] and [Fig Ch1.F4]). This observation may help rationalize why the maximum SE-DNP enhancement in liquids, e.g., about 50 for trityl in glycerol at 320 K [Bibr bib1.bibx26], is not negligibly smaller compared to the enhancements that are obtained in the solid state. We also point out that, while reducing the maximum SE intensities in the vicinity of $\pm \omega_{I}$ , the motional broadening substantially increases the intensities at the smaller offsets around the electronic resonance.

Besides the motional broadening, the progression from left to right in the third row of Fig. [Fig Ch1.F4] demonstrates additional g -tensor broadening, which was also present in “solids”. However, now the two SE lines are affected differently by the g -tensor anisotropy, making the profile of $v_{+}/\langle \delta^{2}\rangle$ at slow tumbling rates rather irregular.

Moving on to the second row in Fig. [Fig Ch1.F4], we see that the SE lines that make up $v_{-}/\langle \delta^{2}\rangle$ (orange) are now completely different from their counterparts in $v_{+}/\langle \delta^{2}\rangle$ (dashed red). The increased intensity in the vicinity of the electronic resonance due to motional broadening is also manifested by $v_{-}/\langle \delta^{2}\rangle$ . For the fastest tumbling in the figure (leftmost column), the fluctuations of the dipolar interaction not only broaden the SE lines, but also enable a new phenomenon, which is manifested as near-resonance peaks that are comparable in magnitude to the peaks at $\pm \omega_{I}$ but are clearly distinct from them (orange line). These peaks reflect the multiplicative contribution of the dispersive EPR signal to $v_{-}$ [Bibr bib1.bibx44]. For faster translational diffusion, the near-resonance peaks may become larger than the peaks at $\pm \omega_{I}$ , as can be seen in the leftmost column of Fig. [Fig App1.Ch1.S1.F10]a (orange line). Because they are more strongly suppressed by the electronic saturation, however, these peaks do not exceed the SE peaks in the final enhancement profile (Fig. [Fig App1.Ch1.S1.F10]a, leftmost column, green line).

Up to an overall scaling factor, the green lines in the last row of Fig. [Fig Ch1.F4] correspond to the SE-DNP enhancement profile. Because its middle part is suppressed by the electronic saturation, this profile in the presence of g -tensor broadening becomes very non-symmetric and responds sensitively to the tumbling of the polarizing agent. To further illustrate the influence of the electronic saturation on the SE-DNP spectrum, in Fig. [Fig App1.Ch1.S1.F9] we show the same curves but calculated with a 5-fold longer electronic spin-lattice relaxation time ( $T_{1}=500$ ns), which leads to larger saturation. Similarly, to illustrate the effect of translational diffusion, we recalculated the curves in Fig. [Fig Ch1.F4] for $\tau_{\mathrm{ffhs}}=3$ ns (2 times faster) and $\tau_{\mathrm{ffhs}}=12$ ns (2 times slower). The results are presented in Fig. [Fig App1.Ch1.S1.F10]. These additional simulations show that the SE-DNP line shape is very sensitive to the timescales of molecular motion.

In the next section, we systematically vary the degrees of power broadening and motional broadening to match the experimental DNP profiles from Fig. [Fig Ch1.F1].

## 5 Disentangling the solid and Overhauser DNP effects

Using the developed methodology, we now analyze the experiments from Fig. [Fig Ch1.F1]. In the light of Eqs. ([Disp-formula Ch1.E2]) and ([Disp-formula Ch1.E7]) for the OE and SE enhancements, we will identify the profile of the electronic saturation (Fig. [Fig Ch1.F4], first row) with $\epsilon_{\mathrm{OE}}$ and the profile of $pv_{-}/\langle \delta^{2}\rangle$ (Fig. [Fig Ch1.F4], last row, green line) with $\epsilon_{\mathrm{SE}}$ . The tumbling times to be used in the DNP calculations will be obtained by fitting the experimental cw-EPR spectra. We start with 10-Doxyl-PC (Fig. [Fig Ch1.F1]a, c) as its experimental spectra were more amenable to unrestricted fits of all parameters.

### 5.1 Analysis of 10-Doxyl-PC

#### 5.1.1 Fit to the cw-EPR spectrum

Derivative EPR spectra were calculated from the first (i.e., 00th) components of the expressions in Eq. ([Disp-formula Ch1.E77]) for different values of the fitting parameters. In the fit, we varied the timescale of tumbling, $\tau_{\mathrm{rot}}$ , as well as the g -tensor anisotropies $\gamma_{0}^{2}$ and $\gamma_{2}^{2}$ (Eq. [Disp-formula Ch1.E50]). As we have no precise knowledge of the field $B_{0}$ at the sample, we freely shifted the calculated spectra along the horizontal axis to achieve the best match with the experiment. Since this leaves one of the g -tensor components undetermined, we took $g_{zz}=2.0023$ , which is typical for nitroxides.

The numerical integrals of the derivative EPR spectra in Fig. [Fig Ch1.F1]a and [Fig Ch1.F1]b (dotted–dashed blue lines) do not come down exactly to zero at the end of the integration range at high-frequency offsets. This points to the possibility that the in-phase component, $s_{y}$ , is mixed slightly with the out-of-phase component, $s_{x}$ . To account for this possibility, we fitted the derivative EPR spectra by calculating

96 $$\frac{\partial s_{y}^{00}}{\partial \Delta }\mathrm{cos}\phi +\frac{\partial s_{x}^{00}}{\partial \Delta }\mathrm{sin}\phi ,$$

where the angle ϕ controlled the degree of mixing.

All in all, not counting the shift along the horizontal axis, we had four fitting parameters: $\gamma_{0}^{2}$ , $\gamma_{2}^{2}$ , $\tau_{\mathrm{rot}}$ and ϕ . The best fit to the cw-EPR spectrum of 10-Doxyl-PC is shown in Fig. [Fig Ch1.F5]a. The corresponding fitting parameters are given in the upper half of Table [Table Ch1.T1].

Encouragingly, our fitted spectrum shows rather good agreement with the experiment, in spite of the simplifying assumptions of the theoretical model, i.e., isotropic rotational diffusion and the absence of hyperfine interaction. To check the effect of the latter on the cw-EPR spectrum, we used Easyspin [Bibr bib1.bibx49] to simulate spectra with our fitted parameters but now also including a nitroxide hyperfine tensor, A=diag(14,14,90) MHz. The result is given in Fig. [Fig App1.Ch1.S1.F11]a. The modification due to the hyperfine interaction, although small as expected at high magnetic fields, is clearly visible. Nevertheless, the comparison of the integrals of the cw-EPR spectra in Fig. [Fig App1.Ch1.S1.F11]b suggests that the error made by neglecting the hyperfine interaction when calculating the DNP spectrum should be small.

Regarding the values of the fitted parameters, it was encouraging to see that the fit resulted in a negligibly small mixing angle of $\phi =-1.3{}^{\circ }$ , indicating that the measured spectrum correctly reflects the in-phase EPR component. With $B_{0}=9.4029$ T and $g_{zz}=2.0023$ , the fitted g -tensor anisotropies that are given in Table [Table Ch1.T1] implied that

97 $$g_{xx}=2.00755,\;g_{zz}=2.00555.$$

These values are rather reasonable for a nitroxide spin label. Finally, the fitted timescale of rotational diffusion was $\tau_{\mathrm{rot}}=5.2$ ns. For comparison, the same timescale for the nitroxide free radical TEMPOL in water is about 20 ps [Bibr bib1.bibx47]. However, unlike TEMPOL, our spin label is covalently attached to the lipid chain.

**Table 1 Ch1.T1:** Parameters obtained from the fits to the experimental data. $B_{1}=0.02$ G for EPR and 5.5 G for DNP. $T_{2}=20$ ns was used for both EPR and DNP.

Fit	Parameter	10-Doxyl-PC	16-Doxyl-PC	
EPR	$\gamma_{0}^{2},\gamma_{2}^{2}$ (MHz)	-373 , 107	From 10-PC	
	$\tau_{\mathrm{rot}}$ (ns)	5.2	1.9	
	ϕ ( ${}^{\circ }$ )	-1.3	-2	
Shown in		Fig. [Fig Ch1.F5]a	Fig. [Fig Ch1.F6]a	
DNP	$\tau_{\mathrm{ffhs}}$ (ns)	6.4	From 10-PC	15.3
	$T_{1}$ (ns)	123	153	141
	$\sigma_{\mathrm{OE}}$ (–)	2.43	2.385	2.57
	$\sigma_{\mathrm{SE}}$ (ps ${}^{-1}$ )	1.51	1.35	1.09
Shown in		Fig. [Fig Ch1.F5]b	Fig. [Fig Ch1.F6]b	Fig. [Fig Ch1.F7]

#### 5.1.2 Fit to the DNP spectrum

Fixing the g -tensor components and the tumbling time to the values obtained from the fit to the cw-EPR spectrum, we proceeded to fit the DNP spectrum of 10-Doxyl-PC (Fig. [Fig Ch1.F1]c). In the calculations, we fixed the mw field to $B_{1}=5.5$ G, which is our best estimate for the home-built Fabry–Pérot resonator operating at maximum power [Bibr bib1.bibx9]. During the fits, we again allowed for global shift of the calculation along the horizontal axis. In addition, we fitted the electronic $T_{1}$ time, which has a direct effect on the electronic saturation profile, as well as the timescale of translational diffusion, $\tau_{\mathrm{ffhs}}$ , which is responsible for the motional broadening of the SE lines.

In the fit, we calculated the electronic saturation factor (Eq. [Disp-formula Ch1.E75]) and the timescale $pv_{-}(\Delta )/\langle \delta^{2}\rangle$ (last equality in Eq. [Disp-formula Ch1.E94]) as functions of the offset frequency Δ . Up to unknown multiplicative factors, these correspond to, respectively, the OE and SE enhancement profiles (Eqs. [Disp-formula Ch1.E2] and [Disp-formula Ch1.E7]). We then fit the experimental DNP spectrum by calculating

98 $$\epsilon (\Delta )=\sigma_{\mathrm{OE}}\times s(\Delta )+\sigma_{\mathrm{SE}}\times \frac{pv_{-}}{\langle \delta^{2}\rangle }(\Delta ),$$

where the scaling parameters $\sigma_{\mathrm{OE}}$ and $\sigma_{\mathrm{SE}}$ were also allowed to vary freely. As a result, not counting the shift along the horizontal axis, our fit contained four fitting parameters: $\tau_{\mathrm{ffhs}}$ , $T_{1}$ , $\sigma_{\mathrm{OE}}$ and $\sigma_{\mathrm{SE}}$ . The best fit to the DNP spectrum of 10-Doxyl-PC is shown in Fig. [Fig Ch1.F5]b. It is noteworthy how the total DNP enhancement (dashed black line) emerges from the sum of the SE (green line) and OE (dotted–dashed blue line) contributions. The corresponding fitting parameters are given in the bottom half of Table [Table Ch1.T1].

In the case of 10-Doxyl-PC, the intuitive analysis of [Bibr bib1.bibx36] for identifying the OE and SE components of a mixed DNP spectrum using the integrated cw-EPR line shape already performed very well (Fig. [Fig Ch1.F1]c). It is, therefore, not surprising that our analysis, which has more fitting parameters, agrees better with the experimental DNP spectrum (Fig. [Fig Ch1.F5]b). Both deficiencies of the intuitive approach, i.e., too narrow OE and SE contributions due to the lack of, respectively, power broadening and motional broadening, appear to be satisfactorily addressed.

On a more fundamental level, our simulation shows that, due to the simultaneous power and motional broadening, the OE and SE contributions to the DNP enhancement are not only rather asymmetric, but also overlap extensively. It should, therefore, be practically impossible to extract any molecular information from the mixed DNP spectrum without a complex, quantitative analysis. In our specific case, the fit resulted in a translational timescale $\tau_{\mathrm{ffhs}}=6.4$ ns and suggested that the electronic relaxation time should be about $T_{1}=120$ ns. At the high magnetic field of the experiment ( $B_{0}=9.4$ T), this spin-lattice relaxation time is practically impossible to measure in the liquid state.

In addition to $\tau_{\mathrm{ffhs}}$ and $T_{1}$ , the fit to the DNP spectrum of 10-Doxyl-PC also produced the following numerical values for the two scaling parameters in Eq. ([Disp-formula Ch1.E98]): $\sigma_{\mathrm{OE}}=2.4$ and $\sigma_{\mathrm{SE}}=1.5$ ps ${}^{-1}$ . These will be analyzed in Sect. [Sec Ch1.S5.SS3] together with the corresponding values for 16-Doxyl-PC.

### 5.2 Analysis of 16-Doxyl-PC

Because the g -tensor anisotropies are largely averaged in the cw-EPR spectrum of 16-Doxyl-PC (Fig. [Fig Ch1.F1]b), we did not attempt to fit them. Instead, we fixed all three components to the values obtained from 10-Doxyl-PC. This left only the rotational time, $\tau_{\mathrm{rot}}$ , and the mixing angle, ϕ , as fitting parameters, not counting the shift along the horizontal axis. As the automated fitting did not behave well, we varied these two parameters manually. One satisfactory fit, obtained with the parameters that are given in Table [Table Ch1.T1], is shown in Fig. [Fig Ch1.F6]a. We mention that the relative heights of the two lines in the calculation were slightly improved by using a small mixing angle of $\phi =-2{}^{\circ }$ .

Although, overall, the fit is not bad, the middle part of the calculated spectrum changes too sharply, and its high-frequency line is too narrow compared to the experiment. We again used Easyspin to check whether these deficiencies are due to the lack of hyperfine interaction. The spectra for $\tau_{\mathrm{rot}}=1.9$ ns with and without hyperfine interaction are shown in Fig. [Fig App1.Ch1.S1.F12]a. As the whole spectrum is narrower than that of 10-Doxyl-PC, the effect of the hyperfine tensor is comparatively larger. Nonetheless, the integrated EPR lines in Fig. [Fig App1.Ch1.S1.F12]b show that the extra width due to the hyperfine tensor should not compromise our subsequent analysis of the DNP spectrum, which will experience additional power broadening and motional broadening.

Moving on to the DNP spectrum, we observed that the free fit of all the parameters resulted in a $\tau_{\mathrm{ffhs}}$ that was more than 2 times larger than that of 10-Doxyl-PC, as we explain below. Considering this to be unrealistic, we fixed $\tau_{\mathrm{ffhs}}$ to the value that was obtained from 10-Doxyl-PC. Thus, not counting the horizontal translation of the calculated DNP spectrum, our automated fit had three fitting parameters: $T_{1}$ , $\sigma_{\mathrm{OE}}$ and $\sigma_{\mathrm{SE}}$ . The outcome is shown in Fig. [Fig Ch1.F6]b. The corresponding parameters are given in the second-last column of the lower half of Table [Table Ch1.T1].

**Figure 6 Ch1.F6:**
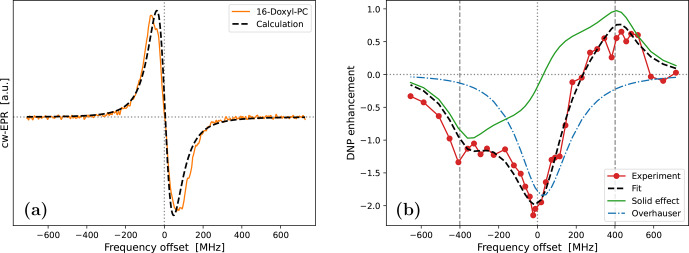
Same as Fig. [Fig Ch1.F5] but for 16-Doxyl-PC. The fitted parameters are given in the second-last column of Table [Table Ch1.T1]. Because all fitted lines in Figs. [Fig Ch1.F5], [Fig Ch1.F6] and [Fig Ch1.F7] are calculated only at the experimental offsets, the green SE lines are not perfectly smooth.

At 9.4 T the electronic Larmor precession timescale is about 0.5 ps, which is 3 orders of magnitude less than the rotational timescales inferred from the cw-EPR spectra. On such sub-picosecond timescales, the local dynamics of the spin labels at positions 10 and 16 should not be very different from each other. Since the spin-lattice relaxation is determined by dynamics on the electronic Larmor timescale, we were satisfied that the fitted $T_{1}=150$ ns was close to that from 10-Doxyl-PC.

The performance of the simple analysis of [Bibr bib1.bibx36] was poorer for 16-Doxyl-PC (Fig. [Fig Ch1.F1]d). Compared to it, our fit to the DNP enhancement profile is excellent (Fig. [Fig Ch1.F6]b). The only parts of the DNP spectrum that our calculation systematically underestimates are the five leftmost experimental points. Although there are other individual experimental points that lie further from the calculated spectrum, these five points are persistently lower by about 0.2 enhancement units.

Observe that the downward shift in the fifth experimental point (together with the first four points) produces an enhancement peak at around -400 MHz. The only way our automated fit can create a pronounced peak at this offset is by making the SE contribution (green line) more “solid-like”, i.e., by increasing $\tau_{\mathrm{ffhs}}$ and reducing the motional broadening. (The lower-left corner of Fig. [Fig App1.Ch1.S1.F10]b provides an example of such a more solid-like SE line shape.) We thus identify the systematic displacement of the leftmost five points as responsible for the increase in $\tau_{\mathrm{ffhs}}$ when it is allowed to vary freely during the fit.

The best fit that we obtained when $\tau_{\mathrm{ffhs}}$ was included among the fitting parameters is shown in Fig. [Fig Ch1.F7]. (The resulting fit parameters are given in the last column of the lower half of Table [Table Ch1.T1].) Indeed, with $\tau_{\mathrm{ffhs}}=15.3$ ns, the SE lines (green) have become sharper, and a small enhancement peak at -400 MHz has emerged (dashed black line). Although the enhancement around +400 MHz has been compromised in the process, the overall fit to all the experimental points is improved compared to Fig. [Fig Ch1.F6]b.

**Figure 7 Ch1.F7:**
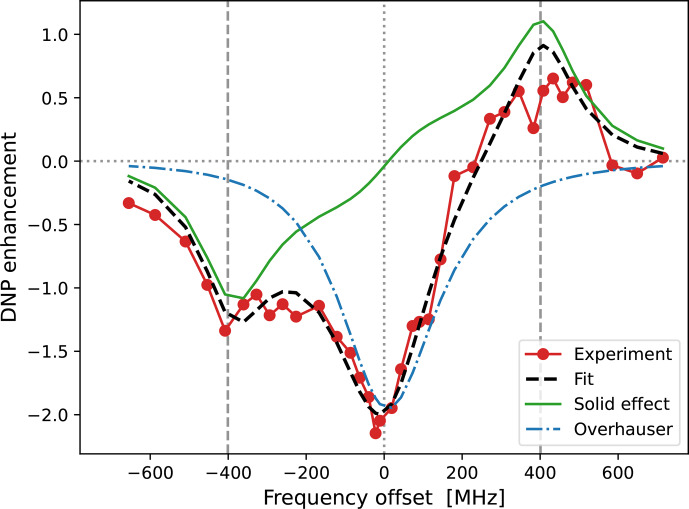
Same as Fig. [Fig Ch1.F6]b but also fitting $\tau_{\mathrm{ffhs}}$ . The fitted parameters are given in the last column of Table [Table Ch1.T1]. Observe that the OE contribution to the DNP spectrum (dotted–dashed blue line) is narrower (i.e., more “liquid-like”) than that of 10-Doxyl-PC (Fig. [Fig Ch1.F5]b), while the SE contribution is more “solid-like” because the timescale $\tau_{\mathrm{ffhs}}$ is 2.4 times longer.

The two alternative fits in Figs. [Fig Ch1.F6]b and [Fig Ch1.F7] correspond to very different timescales of translational diffusion. Nevertheless, within the variability of the measurements, they both agree with the DNP data. Considering the experimental challenges of liquid-state DNP at such high magnetic fields and large mw powers, further decreasing the experimental variability will be very hard. It is, therefore, important to analyze together several different experimental constructs, like our 10- and 16-Doxyl-PC. The final decision of which fit to the DNP spectrum of 16-Doxyl-PC is “better” can only be based on the overall consistency of the fitted parameters across all the analyzed data. We return to this point in Sect. [Sec Ch1.S5.SS3].

The other two parameters that emerged from the fit to the DNP spectrum of 16-Doxyl-PC were $\sigma_{\mathrm{SE}}$ and $\sigma_{\mathrm{OE}}$ . These determine the amplitudes of the SE contribution (solid green lines in Figs. [Fig Ch1.F6]b and [Fig Ch1.F7]) and OE contribution (dotted–dashed blue lines) to the DNP enhancement (dashed black lines). We now turn to the analysis of these scaling parameters.

### 5.3 Additional molecular parameters

Ultimately, the motivation to disentangle a mixed DNP spectrum into its OE and SE components lies in the desire to extract information about the molecular and spin properties that the respective DNP mechanism depends on. The main advantage of our procedure over the intuitive approach of [Bibr bib1.bibx36] is that our decomposition produces physically interpretable parameters like $\tau_{\mathrm{ffhs}}$ and $T_{1}$ . In addition, our scaling parameters $\sigma_{\mathrm{OE}}$ and $\sigma_{\mathrm{SE}}$ multiply, respectively, the saturation factor and $v_{-}/\langle \delta^{2}\rangle$ , whose absolute magnitudes are part of the calculation (Fig. [Fig Ch1.F4], vertical axes). Thus, we can extract further information from the fitted values of $\sigma_{\mathrm{OE}}$ and $\sigma_{\mathrm{SE}}$ . In contrast, because the simple approach rescales the integrated cw-EPR spectrum whose amplitude is arbitrary, the values of its scaling factors are not informative.

Using Eq. ([Disp-formula Ch1.E2]) for the OE enhancement, the coupling factor c is readily expressed in terms of $\sigma_{\mathrm{OE}}$ :

99 $$c=\frac{\sigma_{\mathrm{OE}}}{f}\frac{\gamma_{I}}{\left|\gamma_{S}\right|},$$

where the leakage factor f can be obtained by measuring the nuclear spin-lattice relaxation times (Eq. [Disp-formula Ch1.E4]).

We measured the $T_{1}$ values for the chain protons of DOPC (without spin-labeled lipids) at 310 and 330 K using the Fabry–Pérot probe. These are given in the $T_{1I}^{0}$ column of Table [Table Ch1.T2]. Additionally, we measured the nuclear spin-lattice relaxation times in the presence of either 10- or 16-Doxyl-PC (column $T_{1I}$ of Table [Table Ch1.T2]). The target temperature of the DNP experiments (320 K) lies between the two temperatures at which the nuclear $T_{1}$ times were measured. However, considering the possibility of mild temperature rise by several degrees, we expect the values at 330 K to closely reflect the DNP conditions. Nonetheless, we carry out the following analysis using the $T_{1}$ values measured at both 310 and 330 K.

The leakage factors obtained from Eq. ([Disp-formula Ch1.E4]) are shown in column f of Table [Table Ch1.T2]. Using the values of $\sigma_{\mathrm{OE}}$ from Table [Table Ch1.T1] in Eq. ([Disp-formula Ch1.E99]), we arrived at the coupling factors in column c of Table [Table Ch1.T2]. In the case of 16-Doxyl-PC, the analysis was performed for the fit where $\tau_{\mathrm{ffhs}}$ was fixed at 6.4 ns (denoted as 16 in Table [Table Ch1.T2]) as well as for the fit where $\tau_{\mathrm{ffhs}}$ was free to change (denoted as 16*). (These two alternatives correspond to the last two columns of Table [Table Ch1.T1].) For both choices, somewhat larger coupling factors were deduced for 16-Doxyl-PC compared to 10-Doxyl-PC. The estimated coupling factors are less than 2 times smaller than what we have obtained previously for TEMPOL in DMSO and about 4 times smaller than the coupling factors between TEMPOL and the protons of toluene [Bibr bib1.bibx40].

**Table 2 Ch1.T2:** Analysis of the scaling parameters $\sigma_{\mathrm{OE}}$ and $\sigma_{\mathrm{SE}}$ . Nuclear spin-lattice relaxation times with ( $T_{1I}$ ) and without ( $T_{1I}^{0}$ ) spin labels were measured at two different temperatures. These determine the leakage factor f . The coupling factor c is obtained from f and $\sigma_{\mathrm{OE}}$ using Eq. ([Disp-formula Ch1.E99]). The magnitude of the dipolar interaction responsible for SE ( $\langle \delta^{2}\rangle$ ), obtained from $\sigma_{\mathrm{SE}}$ using Eq. ([Disp-formula Ch1.E100]), provides information about the effective contact distance ( b ). Combining b with $\tau_{\mathrm{ffhs}}$ from Table [Table Ch1.T1], we estimate the diffusion constant of the FFHS model ( $D_{\mathrm{ffhs}}$ ).

	Temperature	Nuclear $T_{1}$ s		Overhauser			Solid effect			
Doxyl-PC	T (K)	$T_{1I}^{0}$ (ms)	$T_{1I}$ (ms)	f	$\sigma_{\mathrm{OE}}$	c (‰)	$\sigma_{\mathrm{SE}}$ (ps ${}^{-1}$ )	$\langle \delta^{2}\rangle T_{1I}$ (ns ${}^{-1}$ )	b (nm)	$D_{\mathrm{ffhs}}$ (nm ${}^{2}$ µ s ${}^{-1}$ )
10	310	580	44	0.92	2.43	3.99	1.51	2.29	0.61	59
	330	910	52	0.94		3.92			0.65	66
16	310	580	93	0.84	2.385	4.32	1.35	2.05	0.81	104
	330	910	120	0.87		4.17			0.89	123
16*	310	580	93	0.84	2.57	4.65	1.09	1.66	0.87	50
	330	910	120	0.87		4.50			0.95	59

Turning now to SE, using the enhancement in Eq. ([Disp-formula Ch1.E7]), we express the unknown strength of the dipolar interaction in terms of the scaling parameter $\sigma_{\mathrm{SE}}$ as follows:

100 $$\langle \delta^{2}\rangle \;T_{1I}=\sigma_{\mathrm{SE}}\frac{\gamma_{I}}{\left|\gamma_{S}\right|}.$$

The values of $\langle \delta^{2}\rangle \;T_{1I}$ , which were calculated from the right-hand side of Eq. ([Disp-formula Ch1.E100]), are about 2 ns ${}^{-1}$ for 10, 16 and 16* (Table [Table Ch1.T2]). Since $v_{+}/\langle \delta^{2}\rangle$ is about 1 ps (Fig. [Fig Ch1.F4], third row), we conclude that $v_{+}T_{1I}\ll 1$ , which justifies our use of the approximation in Eq. ([Disp-formula Ch1.E7]) throughout the analysis, including during the fit to the DNP spectra.

From the expression of $\langle \delta^{2}\rangle$ (Eq. [Disp-formula Ch1.E39]), we can write the contact distance of the translational FFHS model as

101 $$b^{3}=N\frac{2\pi }{5}D_{\mathrm{dip}}^{2}\frac{T_{1I}}{\sigma_{\mathrm{SE}}}\frac{\left|\gamma_{S}\right|}{\gamma_{I}},$$

where N is the number density of the electronic spins. Since, in principle, all parameters on the right-hand side of Eq. ([Disp-formula Ch1.E101]) are measurable, we can determine b . To estimate N , we note that the molecular volume of DOPC is 1.3 nm ${}^{3}$ [Bibr bib1.bibx18]. Since there are 20 unlabeled lipids for 1 labeled one, we estimate N = ( 20×1.3 nm ${}^{3}$ ) ${}^{-1}$ , which corresponds to a molar concentration of 64 mM. Using this number in Eq. ([Disp-formula Ch1.E101]), we obtained the values of b that are given in the second-last column of Table [Table Ch1.T2].

When the values of b are interpreted literally as the “contact distance” between the nitroxide spin label and the protons of the lipid chains, their substantial variation between 10- and 16-Doxyl-PC is disturbing. From that perspective, it is clear that the parameter b of the FFHS model, which we used to account for the fluctuations of the dipolar interaction due to molecular translations, cannot reflect the actual molecular distances of the closest approach.

Because b was obtained from the scaling parameter $\sigma_{\mathrm{SE}}$ , only information about the *amplitude* of the SE enhancement has been directly used in its estimate. In contrast, the motional timescale $\tau_{\mathrm{ffhs}}$ (Table [Table Ch1.T1]) encodes information about the *line shape* of the SE enhancement. From these complementary features of the SE contribution to the DNP spectrum, we have managed to determine both b and $\tau_{\mathrm{ffhs}}$ . Having access to these two parameters, we can calculate the diffusion constant of the FFHS model from Eq. ([Disp-formula Ch1.E38]). The results are given in the last column of Table [Table Ch1.T2]. To our surprise, we obtained very similar values for 10 and 16*, while the diffusion constant for 16 is 2-fold larger. (Given the variability in the experimental data and the fact that the fits to the DNP spectra are not unique, the differences between $D_{\mathrm{ffhs}}$ of 10 and 16* should not be seen as meaningful.)

In an effort to identify a potential candidate for the physical motion that the FFHS model emulates, we observed that the coefficients of lateral translational diffusion for DOPC in oriented bilayers are 20 nm ${}^{2}$ µ s ${}^{-1}$ at 323 K and 26 nm ${}^{2}$ µ s ${}^{-1}$ at 333 K ([Bibr bib1.bibx12], Fig. 6a). These, we expect, will bracket the value under our DNP conditions. The diffusion in the FFHS model corresponds to the relative translation of the nuclear and electronic spins, i.e., $D_{\mathrm{ffhs}}=D_{I}+D_{S}$ . Assuming that the lateral diffusion of spin-labeled PSPC in a DOPC bilayer is similar to that of DOPC, from the measured values given above we would expect $D_{\mathrm{ffhs}}$ to be between 40 and 52 nm ${}^{2}$ µ s ${}^{-1}$ . This range is surprisingly close to the estimates of 10 and 16* in the last column of Table [Table Ch1.T2], which suggests that the FFHS model in our analysis likely accounts for the lateral diffusion of the lipids in the plane of the bilayer.

Since it leads to a diffusion constant that is similar to (i) the known lateral diffusion of DOPC and (ii) the estimate obtained for 10-Doxyl-PC, we conclude that the fit to the DNP spectrum of 16-Doxyl-PC that is shown in Fig. [Fig Ch1.F7] (i.e., the one that led to “unreasonably” large $\tau_{\mathrm{ffhs}}$ ) is more realistic than the one with fixed $\tau_{\mathrm{ffhs}}$ (Fig. [Fig Ch1.F6]b). From the perspective of the diffusion constant, the longer translational timescale of 16* compared to 10, which resulted in a more solid-like SE line shape with less motional broadening, reflects the fact that the “contact distances” in the two cases are different. In retrospect, it is amazing how the independent estimates of b and $\tau_{\mathrm{ffhs}}$ combine to yield practically identical diffusion constants for the two spin-labeling positions.

At the moment, it is not clear to us how to properly interpret the different values of b at positions 10 and 16. Atomistic molecular dynamics simulations [Bibr bib1.bibx37] could, in principle, be used to investigate whether these effective contact distances reflect differences in proton density along the normal of the lipid bilayer or arise for some other reason.

### 5.4 Limitations of the modeling

The calculated DNP spectra of 10-Doxyl-PC (Fig. [Fig Ch1.F5]b) and 16-Doxyl-PC (Fig. [Fig Ch1.F7]) agree well with the experiments in spite of our simplistic treatment of the quantum and classical dynamics. Specifically, when modeling the spin dynamics, (i) we completely neglected the hyperfine interaction with the nuclear spin of ${}^{14}$ N, which is present in nitroxide spin labels. In the case of the classical dynamics, (ii) we modeled the reorientation of the spin labels at positions 10 and 16 of the lipid chain as free, isotropic diffusion, and (iii) we modeled the dynamics of the acyl protons relative to the unpaired electron as isotropic translational diffusion that extends to infinity in all three spatial directions. We now comment on these deficiencies of the modeling.

Starting with the third point, it is clear that the translational diffusion of the polarized aliphatic protons (as well as that of the chain-attached spin labels) must be confined to the interior of the lipid bilayer and should not extend arbitrarily far along the direction perpendicular to the bilayer plane. In contrast, the FFHS model whose analytical correlation function we used in the calculations assumes isotropic diffusion in all spatial directions. To properly address this deficiency of the modeling, one would need to solve the diffusion equation with boundary conditions that reflect the confining planar geometry of the lipid bilayer and then calculate the dipolar correlation function for such confined diffusion (preferably in closed, analytical form). In the meantime, one could argue that, because the dipolar interaction drops rapidly with distance, an overwhelming contribution to the dipolar correlation function should come from configurations in which the electron and nucleus are close to each other. In that case, the unphysical configurations that place the acyl protons outside the plane of the lipid bilayer (but are allowed in the FFHS model) may contribute relatively little. To support this argument, we observe that the numerical values of the FFHS parameter b in Table [Table Ch1.T2] indicate that the shortest relevant distances for SE are about 0.6 nm (for 10-Doxyl-PC) and 0.9 nm (for 16-Doxyl-PC). These are 3 to 5 times smaller than the hydrophobic thickness of the DOPC lipid bilayer, which is about 3 nm [Bibr bib1.bibx23].

Regarding the second deficiency, the problem here is that the nitroxide spin label is covalently fused to the lipid chain; thus, its possible orientations should reflect the preferred alignment of the chain in the hydrophobic core of the bilayer. Furthermore, the fused nitroxide is not expected to have identical diffusion rates for rotations about different spatial directions. Clearly, both of these aspects (i.e., the orientational preference and the anisotropy) are missing from the free, isotropic rotational diffusion that we implemented. It is, however, known how to account for them in a rigorous and efficient way. Indeed, the MOMD model from the Freed lab [Bibr bib1.bibx33] treats anisotropic rotational diffusion in a restoring potential. In fact, this model has been extensively used to simulate high-field cw-EPR spectra of lipid bilayers by Freed [Bibr bib1.bibx32] and Marsh [Bibr bib1.bibx30]. The studies of Marsh and colleagues have focused on DMPC lipid bilayers containing appreciable amounts of cholesterol, which puts them in a liquid-ordered phase. For DMPC with 40 mol % cholesterol at 30 ${}^{\circ }$ C, 10-Doxyl-PC was deduced to be aligned with the director (i.e., the direction normal to the bilayer plane) with an order parameter S=0.67 [Bibr bib1.bibx30]. If the orientational motion is imagined as being confined to a cone [Bibr bib1.bibx29], this order parameter would correspond to a maximum possible deviation from the director of $\theta_{0}=40{}^{\circ }$ in all directions. The lipid bilayers in our experiments are composed of pure DOPC lipids and are in their liquid-crystalline phase, where the ordering is substantially reduced. The liquid-crystalline phase of pure DPPC lipid bilayers has been characterized in the studies of Freed and colleagues. The order parameter reported for 16-Doxyl-PC in pure DPPC at 50 ${}^{\circ }$ C is S=0.16 [Bibr bib1.bibx8]. It corresponds to a maximum possible deviation from the director of $\theta_{0}=75{}^{\circ }$ , assuming the diffusion is confined to a cone. Although 10-Doxyl-PC is expected to be more ordered than 16-Doxyl-PC, it is not clear how much smaller than $\theta_{0}=75{}^{\circ }$ its corresponding cone angle would be. (Because S is the expectation value of a rank-2 spherical harmonic, the free rotational diffusion that we use corresponds to $\theta_{0}=90{}^{\circ }$ .) From these studies we conclude that the MOMD model (with an axial diffusion tensor) will likely improve our fits to the experimental cw-EPR spectra. Nevertheless, free rotation may still be a good first approximation to the orientational dynamics of 10- and 16-Doxyl-PC in the liquid-crystalline phase of our lipid bilayers.

We should emphasize that our aim in the current paper is to show how to account for the rotational dynamics of the polarizing agent in the calculation of SE-DNP. In this context, we observe that, while the cw-EPR spectra in derivative mode (Figs. [Fig App1.Ch1.S1.F11]a and [Fig App1.Ch1.S1.F12]a) are extremely sensitive to the details of the rotational motion of the radical, their integrals (Figs. [Fig App1.Ch1.S1.F11]b and [Fig App1.Ch1.S1.F12]b) are much more forgiving. When contributing to the DNP spectrum, these integrated EPR line shapes are additionally broadened by mw power (OE) and translational diffusion (SE) (Figs. [Fig Ch1.F5]b and [Fig Ch1.F7]). All these factors are expected to reduce the sensitivity of the DNP spectrum to the details of the radical tumbling (at least in comparison to the sensitivity of the cw-EPR line shape). We therefore think that, for the purposes of fitting the DNP spectrum, further improving the fit to the cw-EPR spectra at the cost of introducing more fitting parameters is not really justified. That being said, we stress that the formalism of Sect. [Sec Ch1.S4] can be straightforwardly extended to anisotropic diffusion in an orienting potential (i.e., the MOMD model). This would lead to larger matrices $R_{1}$ , $R_{2}$ and Δ , whose matrix elements would be different than the expressions we gave in Sect. [Sec Ch1.S4.SS2] for free, isotropic rotational diffusion.The orienting potential will mix coefficients with different values of L , which will result in non-diagonal $R_{1}$ and $R_{2}$ . The anisotropic rotation will mix coefficients whose M indices differ by ±1 , so odd values of M will also need to be included. Finally, since the potential is defined with respect to the director axis, which may differ from the axes of both the laboratory frame and the molecular frame, it will be necessary to consider coefficients $s_{MN}^{L}$ with non-zero index N . Clearly, for a given $L_{\max}$ , the resulting matrices $R_{1}$ , $R_{2}$ and Δ will be substantially larger. A detailed presentation can be found in [Bibr bib1.bibx42] and [Bibr bib1.bibx39]. Once correctly formed, these three matrices can be directly used in Eqs. ([Disp-formula Ch1.E92]) and ([Disp-formula Ch1.E94]) to calculate the SE-DNP spectra in, respectively, “solids” and liquids.

Moving on to the first deficiency mentioned above, we remind the reader that we describe the SE spin dynamics in terms of two sets of Bloch equations that are connected in series [Bibr bib1.bibx44]. These are the classical Bloch equations with the Bloch matrix $B_{0}$ (Sect. [Sec Ch1.S3.SS1]) and the “new Bloch equations” (Eq. [Disp-formula Ch1.E21]) with the matrix $B=B_{0}+\text{i}\omega_{I}$ (Sect. [Sec Ch1.S3.SS2]). Because our description of SE-DNP is based on Bloch equations, in Sect. [Sec Ch1.S4.SS1] and [Sec Ch1.S4.SS2] we reformulated Freed's treatment of slow tumbling as a generalization of the classical Bloch equations, such that the scalar elements of $B_{0}$ became matrices in the space of the angular-momentum indices LM . The result was the “expanded Bloch matrix” $B_{0}$ in Eq. ([Disp-formula Ch1.E63]). For this reformulation to work, however, we had to neglect the hyperfine interaction, which is in fact treated by [Bibr bib1.bibx14]. As a result, our analysis is formally deficient for nitroxide radicals. Nevertheless, we reasoned that it should be possible to illustrate the theoretical formalism in its current form by focusing on nitroxides at high magnetic fields, where the hyperfine interaction is expected to be negligible compared to the anisotropy of the g tensor. From this perspective, it should be clear that the DNP experiments that we analyzed here had been carefully selected.

Figures [Fig App1.Ch1.S1.F11] and [Fig App1.Ch1.S1.F12] show our attempt to assess the contribution of the neglected hyperfine interaction to the (integrated) EPR spectra of 10- and 16-Doxyl-PC. A somewhat more detailed analysis is contained in our response to the reviewers, which is freely accessible online. There we observe that the hyperfine interaction slightly broadens the EPR line of 16-Doxyl-PC (which is also visible in Fig. [Fig App1.Ch1.S1.F12]a). Since the only mechanism of broadening in our case is the rotational tumbling, our choice of $\tau_{\mathrm{rot}}=1.9$ ns (Table [Table Ch1.T1]) likely compensates for some of the “missing” hyperfine broadening. Such compensation does not appear to be happening in the case of 10-Doxyl-PC, where the hyperfine interaction changes the shape but not the width of the EPR line (Fig. [Fig App1.Ch1.S1.F11]a). Ultimately, for the theory to be applicable to SE-DNP with nitroxide polarizing agents at lower magnetic fields, like X band [Bibr bib1.bibx15], the description of the spin dynamics will need to include the nuclear spin of ${}^{14}$ N. Since the dimension of the resulting Liouville space would need to increase by a factor of 9, it should be possible to preserve the two sets of connected Bloch equations after replacing each of their scalar matrix elements with a 9×9 matrix. Alternatively, the two sets of Bloch equations should be replaced with the corresponding equations of motion for the density matrix in Liouville space. However, considering the inherent experimental uncertainty of the DNP enhancements that we compare with (Fig. [Fig Ch1.F1]c and [Fig Ch1.F1]d) and the achieved agreement between simulation and experiment (Figs. [Fig Ch1.F5]b and [Fig Ch1.F7]), we believe that such more complex modeling is presently not justified.

## 6 Conclusion

Once the spin dynamics of the solid effect has been formulated in the time domain [Bibr bib1.bibx44], it becomes possible to interface this quantum dynamics with various types of classical dynamics. The classical dynamics in [Bibr bib1.bibx45] was the translational diffusion of the spins in a liquid; here we additionally included the rotational diffusion of the polarizing agent. To illustrate the practical utility of the resulting formalism, we analyzed either previously published [Bibr bib1.bibx45] or previously unpublished (current paper) experimental DNP data on lipid bilayers. In our analysis, the treatment of molecular translation and rotation was limited to the simplest possible models of free, isotropic diffusion. Surprisingly, in spite of the spatial anisotropy that one expects for hydrated lipid bilayers, previously we found that isotropic translation, as described by the FFHS model, worked well for the free radical BDPA in DMPC bilayers [Bibr bib1.bibx45]. Similarly, in the current paper we found that the simplest treatment of free, isotropic rotation (together with FFHS translation) reproduced well the DNP field profiles of nitroxide-labeled lipids in DOPC bilayers.

DNP experiments with nitroxide free radicals in viscous liquids invariably manifest a mixture of SE and OE [Bibr bib1.bibx28]. As these two DNP mechanisms are sensitive to molecular motions on vastly different timescales, it should be possible to obtain rich dynamical information by analyzing their contributions to the overall DNP enhancement. Disentangling the SE and OE contributions, however, has proven to be challenging [Bibr bib1.bibx27]. Here we fitted liquid-state DNP spectra by calculating enhancements that were affected by both the translational diffusion of the spins and the rotational diffusion of the free radical. Since different motions modify the amplitude and the shape of the DNP spectrum in a highly concerted manner, by fitting the entire line shape of the enhancement, we also gained access to the absolute magnitudes of the SE and OE contributions.

Our current treatment of SE-DNP in liquids uses only the correlation function of the dipolar interaction to describe the translational motion of the spins [Bibr bib1.bibx45]. This is formally correct only when the diffusion is much faster than the nuclear $T_{1}$ relaxation. It should be possible to relax this condition and model slower spin diffusion, as relevant for SE in the solid state.

## Figures and Tables

**Figure A1 App1.Ch1.S1.F8:**
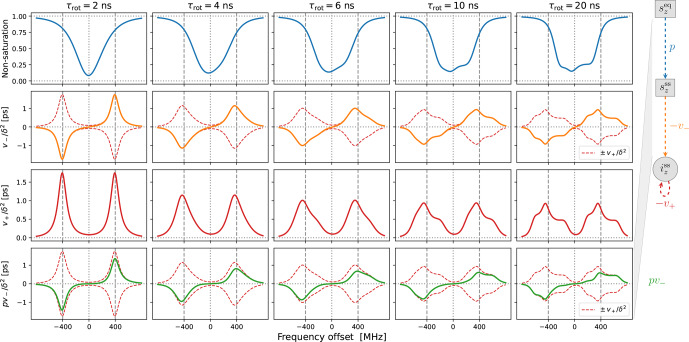
Same as Fig. [Fig Ch1.F3] but with $T_{1}=500$ ns (i.e., 5-fold longer), which leads to larger saturation of the allowed electronic transition. Only the first and last rows are affected.

**Figure A2 App1.Ch1.S1.F9:**
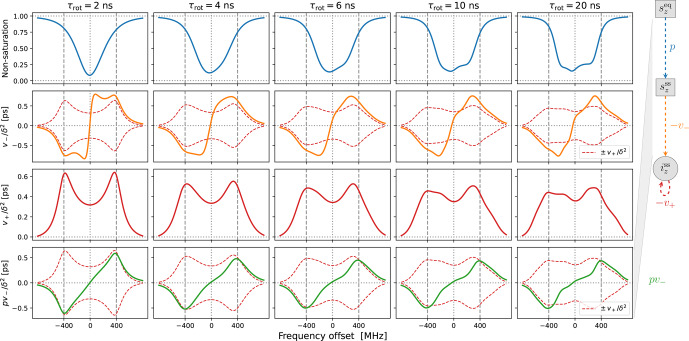
Same as Fig. [Fig Ch1.F4] but with $T_{1}=500$ ns, which leads to larger electronic saturation. As in the case of “solids”, only the first and last rows are affected.

**Figure A3 App1.Ch1.S1.F10:**
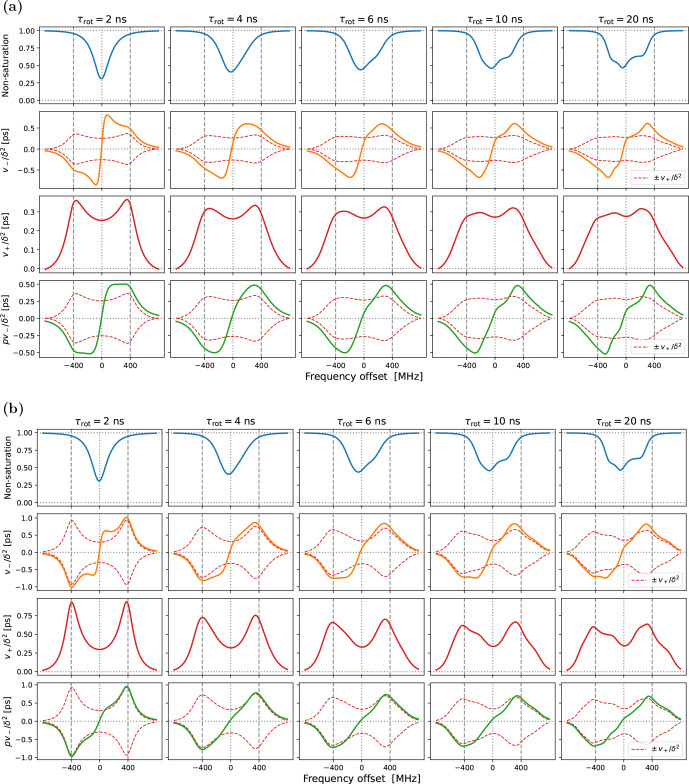
Same as Fig. [Fig Ch1.F4] but with **(a)** $\tau_{\mathrm{ffhs}}=3$ ns, i.e., 2-fold faster translational motion which broadens the SE lines to a larger extent, and **(b)** $\tau_{\mathrm{ffhs}}=12$ ns, i.e., more solid-like behavior. Observe how the predicted SE-DNP line shape (green line in the last row) responds sensitively to the timescale of the translational motion that is responsible for averaging the dipolar interaction.

**Figure A4 App1.Ch1.S1.F11:**
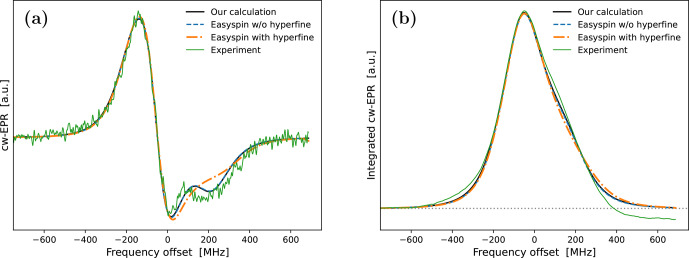
Effect of the hyperfine tensor on the calculated EPR spectrum of 10-Doxyl-PC. Various derivative cw-EPR spectra **(a)** and their numerical integrals **(b)** are compared with each other. Our calculation (solid black line) agrees perfectly with the Easyspin [Bibr bib1.bibx49] simulation without a hyperfine tensor (dashed blue line). Including a hyperfine tensor with components (14,14,90) MHz in the Easyspin calculation (dotted–dashed orange line) leads to visible changes in the derivative cw-EPR spectrum. However, the difference of the integrated EPR lines with and without a hyperfine tensor in panel **(b)** should be negligible as far as the simulation of the DNP spectrum is concerned.

**Figure A5 App1.Ch1.S1.F12:**
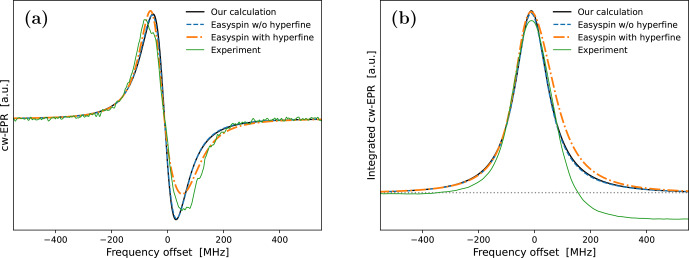
Same as Fig. [Fig App1.Ch1.S1.F11] but for 16-Doxyl-PC. Because the cw-EPR spectrum is narrower to begin with, the relative contribution of the hyperfine tensor with components (14,14,90) MHz is larger than in the case of 10-Doxyl-PC. Considering that the EPR line will experience additional power broadening and motional broadening in DNP, it should still be possible to safely neglect the extra width that the hyperfine tensor brings to the integrated EPR line in panel **(b)**.

## Data Availability

The analyzed experimental data and the code used to generate the figures in the paper are available at https://github.com/dzsezer/solidDNPliquids_g-tensor (last access: 23 October 2023) (https://doi.org/10.5281/zenodo.8360325, Sezer, 2023c).
